# How important is the linearity assumption in a sample size calculation for a randomised controlled trial where treatment is anticipated to affect a rate of change?

**DOI:** 10.1186/s12874-023-02093-2

**Published:** 2023-11-21

**Authors:** Katy E. Morgan, Ian R. White, Chris Frost

**Affiliations:** 1 Department of Medical Statistics, London School of Hygiene and Tropical Medicine, London, UK; 2 MRC Clinical Trials Unit at UCL, University College London, London, UK

**Keywords:** Random slopes model, Linearity assumption, Sample size calculation, Randomised clinical trial, Model misspecification

## Abstract

**Background:**

For certain conditions, treatments aim to lessen deterioration over time. A trial outcome could be change in a continuous measure, analysed using a random slopes model with a different slope in each treatment group. A sample size for a trial with a particular schedule of visits (e.g. annually for three years) can be obtained using a two-stage process. First, relevant (co-) variances are estimated from a pre-existing dataset e.g. an observational study conducted in a similar setting. Second, standard formulae are used to calculate sample size. However, the random slopes model assumes linear trajectories with any difference in group means increasing proportionally to follow-up time. The impact of these assumptions failing is unclear.

**Methods:**

We used simulation to assess the impact of a non-linear trajectory and/or non-proportional treatment effect on the proposed trial’s power. We used four trajectories, both linear and non-linear, and simulated observational studies to calculate sample sizes. Trials of this size were then simulated, with treatment effects proportional or non-proportional to time.

**Results:**

For a proportional treatment effect and a trial visit schedule matching the observational study, powers are close to nominal even for non-linear trajectories. However, if the schedule does not match the observational study, powers can be above or below nominal levels, with the extent of this depending on parameters such as the residual error variance. For a non-proportional treatment effect, using a random slopes model can lead to powers far from nominal levels.

**Conclusions:**

If trajectories are suspected to be non-linear, observational data used to inform power calculations should have the same visit schedule as the proposed trial where possible. Additionally, if the treatment effect is expected to be non-proportional, the random slopes model should not be used. A model allowing trajectories to vary freely over time could be used instead, either as a second line analysis method (bearing in mind that power will be lost) or when powering the trial.

**Supplementary Information:**

The online version contains supplementary material available at 10.1186/s12874-023-02093-2.

## Background

In certain diseases, a person’s condition deteriorates over time. Potential treatments for such a disease might aim to lessen the rate of that deterioration. When conducting a trial of a treatment like this, a common choice of outcome is a continuous variable that can capture any deterioration over time, measured on multiple occasions during follow-up. For example, brain atrophy is used as an outcome for clinical trials measuring deterioration in early-stage manifest Huntington’s Disease [1]. Examples from other disease areas include the use of log PD_20_ in asthma patients (example given in [2]), and bone mineral density in osteoporosis patients (example given in [3]).

Such a continuous longitudinal trial outcome is often analysed using a mixed-effects model, with a random intercept and slope over time for each participant to allow for the dependence of measurements taken on the same person. The fixed part of the model includes a slope over time in the control group and a slope over time in the treated group, with the treatment effect being the difference between these two slopes. Thus, an effective treatment will slow the mean rate of deterioration in those receiving the trial treatment compared to participants receiving control.

A trial sample size can be obtained for a random slopes model by using standard sample-size formulae for mixed-effects models [4, 5]. Such calculations depend on the estimated variance of the treatment effect, which in turn depends on the between- and within-subject variances and covariances implied by the random slopes model. It may be hard to obtain likely values for these (co)variances. One source of estimates is any existing dataset collected in a similar population to the proposed trial, for example an observational study conducted in a similar setting. Estimates of the necessary parameters can be obtained by fitting a random slopes model to the existing data, and these estimates can then be used in the sample-size formula. Examples of this methodology being used in practice can be found in a Huntington’s Disease setting in reference [1], in an Alzheimer’s Disease setting in reference [6], and in a Multiple Sclerosis setting in references [7, 8].

The random slopes model outlined above assumes that trajectories in both the treatment and control groups are linear over time, and hence that the difference between the means increases linearly over time, but this may not be the case. Imaging and biomarker changes can be markedly non-linear particularly over long periods of time. For example, there was some evidence that the rate of percentage brain volume loss increased as disease severity progressed in 12 patients with Alzheimer’s disease [9]. It is also possible for cognitive outcomes to have non-linear trajectories. For example, in a phase 2 trial of donanemab in patients with early symptomatic Alzheimer’s disease, the primary outcome of Integrated Alzheimer’s Disease Rating Scale showed very little change early in follow-up, with worsening occurring later in the trial (Fig. 2, panel A, [10]).

In addition to trajectories being non-linear, treatment effects are also sometimes found to be non-linear in time. For example, the SmaRT Oncology-2 trial examined an integrated treatment programme for major depression in oncology patients versus usual care. Instead of a steady improvement over the course of the trial, the intervention improved depression scores early in the trial, and this effect was then sustained to the end of follow-up (Fig. 2, [11]). Another example, again from the phase 2 trial of donanemab in Alzheimer’s patients, can be seen in the secondary outcome amyloid plaque in which treatment resulted in an initial rapid decrease in plaque levels that then levelled off later in follow-up (Fig. 3, [10]).

The primary aim of this paper is therefore to assess the impact of assuming that outcomes evolve linearly over time when in truth they do not, in terms of specifying the sample size for a future trial from an existing dataset. We use a simulation study and consider scenarios in which the outcome evolves non-linearly over time but the treatment effect on the mean is still proportional to time, and those in which both the trajectory and treatment effect deviate from the assumed linear model.

It is possible that investigators might suspect at the design stage that there are non-linear trajectories, but not have sufficient evidence of this in the previously collected dataset. These non-linearities might become more apparent in the trial data. For example, in the AIMS trial [12], which examined the effect of irbesartan in Marfan syndrome, the primary analysis specified a random slopes model with the aortic diameter outcome changing linearly with time. However, it was necessary to include a sensitivity analysis in which the linearity assumption was removed, since trajectories were found to be non-linear. In addition, it can be very difficult to accurately predict the shape of the treatment effect for a new treatment in advance. If there is strong reason to suspect a non-proportional treatment effect, then a different model may need to be used for the sample size calculation. But in other situations, it might be sufficient to add a secondary analysis method to the statistical analysis plan for the trial to guard against these possibilities. The secondary aim of this paper is therefore to compare the performance of the random slopes model with three alternative analysis models in terms of the treatment effect and standard error estimated, as well as the power achieved. When the underlying assumptions hold, the random slopes model will be more statistically efficient than less restrictive models. However, when the assumptions do not hold, less restrictive models may be necessary to obtain correct estimates of the treatment effect.

In the next section, we outline the sample-size methodology used. We then go on to describe our simulation study in the "Simulation study" section, including the data generation mechanism and the scenarios considered, and give our results in the "Results" section. We finish by giving a discussion and conclusion of our results.

## Methods

In this section, we give a brief overview of the existing sample-size methodology used in this paper. A fuller explanation of this can be found in [1, 4, 5, 13]. We go on to outline some possible alternative methods of analysis in the "Possible alternative models for analysis" section.

### Analysis model assumed at design stage

Let us consider a setting in which a continuous outcome is measured multiple times during follow-up and is modelled using the following random slopes model in the trial analysis:

1 $$\begin{array}{c}{y}_{ij}={\beta }_{0}+{\beta }_{1}{t}_{j}+\gamma {z}_{i}{t}_{j}+{u}_{0i}+{u}_{1i}{t}_{j}+{e}_{ij}\end{array}$$

where ${y}_{ij}$ is the outcome for the ${i}^{th}$ person at the ${j}^{th}$ visit, for $i=1,\dots ,{N}_{trial}$ and $j=0,\dots ,{n}_{t}$; ${t}_{j}$ is the time of the ${j}^{th}$ visit and ${t}_{0}=0$ at baseline; ${z}_{i}$ is 1 if the ${i}^{th}$ person is in the treated group and 0 if they are in the control group. Therefore, ${\beta }_{0}$ is the expected outcome in both treatment groups at baseline, ${\beta }_{1}$ is the mean slope over time in the control group, and $\gamma$ is the difference between the mean slope in the treated group and that in the control group. The use of a common mean level at baseline, ${\beta }_{0}$, takes advantage of the fact that the trial is randomised, and therefore that the two groups are on average the same at the start of the trial. This constraint results in a more efficient analysis compared to allowing a separate expected value for each group at baseline [14]. In this model $\gamma$ is the treatment effect on the slope. Since we assume that there is no difference between the treatment groups at baseline, we can also express the treatment effect as $\gamma \times {t}_{{n}_{t}}$, the difference between the means in the two treatment groups at the final time point of the trial. Re-parameterising the treatment effect in this way allows us to compare it with treatment effects from other models (introduced in the "Possible alternative models for analysis" section, and further discussed in the "Estimand of interest" section). ${u}_{0i}$ and ${u}_{1i}$ are random intercepts and slopes for person $i$ respectively, and ${e}_{ij}$ are residual errors. All are assumed to be normally distributed as follows:

2 $$\begin{array}{c}\left(\begin{array}{c}{u}_{0i}\\ {u}_{1i}\end{array}\right)\sim N\left[\left(\begin{array}{c}0\\ 0\end{array}\right),\left(\begin{array}{cc}{\sigma }_{u0}^{2}& {\sigma }_{u01}\\ {\sigma }_{u01}& {\sigma }_{u1}^{2}\end{array}\right)\right]; {e}_{ij}\sim N\left[0,{\sigma }_{e}^{2}\right]\end{array}$$

where ${\sigma }_{u0}^{2}$ and ${\sigma }_{u1}^{2}$ are the variances for the random intercepts and slopes respectively, and ${\sigma }_{u01}$ is the covariance between them.

A mixed-effects model such as the one in Eq. (1) can be written in general matrix form:

$${\varvec{Y}}|{\varvec{u}}\sim N\left[{\varvec{X}}{\varvec{\beta}}+{\varvec{Z}}{\varvec{u}},{\varvec{R}}\right]$$

where ${\varvec{Y}}$ is a vector of all ${N}_{trial}\times {n}_{t}$ outcomes, ${\varvec{X}}$ and ${\varvec{Z}}$ are design matrices for the fixed and random effects respectively, ${\varvec{\beta}}={\left({\beta }_{0},{\beta }_{1},\gamma \right)}^{T}$ and ${\varvec{u}}$ are vectors of the fixed and random effects respectively, with ${\varvec{u}}\sim N\left[0, {\varvec{G}}\right]$ where ${\varvec{G}}$ is block diagonal with the covariance matrix from (2) on the diagonal, and ${\varvec{R}}$ is a diagonal matrix with ${\sigma }_{e}^{2}$ on the diagonal.

We have introduced the random slopes model in its conditional form, but we can also write it in marginal form. Since our outcome is continuous, the treatment effects and standard errors will be identical from the conditional and marginal formulations, and considering the marginal form allows us to use standard matrix formulae for obtaining the standard error (SE) of the treatment effect. Writing this model in marginal form we get:

$${\varvec{Y}}\sim N\left[{\varvec{X}}{\varvec{\beta}},\boldsymbol{\Sigma }={\varvec{R}}+{\varvec{Z}}{\varvec{G}}{{\varvec{Z}}}^{T}\right]$$

Standard theory [15] gives the following matrix equation for the fixed effects:

$$\widehat{{\varvec{\beta}}}={\left({{\varvec{X}}}^{T}{{\varvec{\Sigma}}}^{-1}{\varvec{X}}\right)}^{-1}{{\varvec{X}}}^{T}{{\varvec{\Sigma}}}^{-1}{\varvec{Y}}$$

### Sample size calculation

To calculate a sample size for a trial analysed using the model specified in (1), it is necessary to find the variance of the treatment effect estimated from the random slopes model. Again, from standard theory [15], the variance of $\widehat{\gamma }$ can be obtained (along with the variances for the other fixed effects) from:

3 $$\begin{array}{c}V\left(\widehat{{\varvec{\beta}}}\right)={\left({{\varvec{X}}}^{T}{\boldsymbol{\Sigma }}^{-1}{\varvec{X}}\right)}^{-1}\end{array}$$

The relevant entry of this variance–covariance matrix is the 3 × 3^th^ element – the variance of the treatment effect, $V\left(\widehat{\gamma }\right)$. If we calculate this quantity for a hypothetical 2-person trial (with one person in the control arm and one in the treatment arm), and assuming equal allocation, a sample size for testing the null hypothesis that $\gamma =0$ from the analysis model in (1) can then be calculated using:

4 $$\begin{array}{c}N=2\times \left[{\frac{{\left({z}_{1-\frac{\alpha }{2}}+{z}_{1-\beta }\right)}^{2}}{{d}^{2}}}V\left(\widehat{\gamma }\right)\right]\end{array}$$

where the contents of the square brackets should be rounded up to the nearest integer, $d$ is the target treatment effect (parameterised as a difference in slopes), $\alpha$ is the Type I error, and $1-\beta$ is the power. The variance from a 2-person trial is used as a computational simplification since the variance for an $N$ person trial is $\frac{V\left(\widehat{\gamma }\right)}{N/2}$ – see [4] for details.

To get $V\left(\widehat{\gamma }\right)$ from (3) (or equivalently from the non-matrix formulation of this expression in reference [5]), and hence calculate $N$, it is necessary to construct the $2{n}_{t}\times 2{n}_{t}$ matrix $\boldsymbol{\Sigma }$ for this hypothetical 2-person trial, and we therefore need estimates of the parameters within $\boldsymbol{\Sigma }$, i.e., ${\sigma }_{u0}^{2},{\sigma }_{u1}^{2},{\sigma }_{u01}$ and ${\sigma }_{e}^{2}$. These parameters can be estimated from an existing dataset; for example, an observational study measuring outcomes on people with the disease but who are not receiving the trial treatment. The observational study can be analysed using a simpler version of the random slopes model in (1) that removes the term involving $\gamma$ from the fixed part of the model:

5 $$\begin{array}{c}{y}_{ij}={\beta }_{0}+{\beta }_{1}{t}_{j}+{u}_{0i}+{u}_{1i}{t}_{j}+{e}_{ij}\end{array}$$

with ${u}_{0i}, {u}_{1i}$ and ${e}_{ij}$ distributed as in (2). Since the random slopes model assumes a particular covariance structure over time, we can use the parameter estimates to calculate sample sizes for trials with different visit schedules than observed in the observational study.

The Stata package slopepower implements this sample size calculation given an existing dataset [13]. A shiny app [16] written by Hu et al. provides similar functionality using R, although without the first step process of fitting the random slopes model to an existing dataset. Instead, users input the values of the relevant parameters having obtained these values themselves.

### Possible alternative models for analysis

In the previous section, we described the random slopes model (Eqs. (1) and (2)) that can be used to analyse the sort of trial we are considering. The random slopes model assumes linear trajectories in both the control and treated groups, and hence a treatment effect that increases linearly over time. However, as discussed in the introduction, these assumptions may not hold, in which case a less restrictive analysis model might be needed. We therefore consider several models that relax the assumptions of the random slopes model, both in terms of the mean trajectories and in terms of the covariance structure. When relaxing the assumptions relating to the covariance structure it is easier to consider the marginal form of the model. Since we restrict to consideration of continuous outcomes, the treatment effects estimated from a participant-conditional model and a corresponding marginal model that averages across participants are numerically equivalent.

One possible alternative is to use a model that has the same mean structure as in (1) but an unstructured covariance matrix:

6 $$\begin{array}{c}{y}_{ij}={\beta }_{0}+{\beta }_{1}{t}_{j}+\gamma {z}_{i}{t}_{j}+{\epsilon }_{ij}\\ {{\varvec{\epsilon}}}_{{\varvec{i}}}=\left(\begin{array}{c}{\epsilon }_{i0}\\ {\epsilon }_{i1}\\ \vdots \end{array}\right)\sim N\left[\left(\begin{array}{c}0\\ 0\\ \vdots \end{array}\right),{\varvec{\Sigma}}=\left(\begin{array}{ccc}{\upsigma }_{00}& {\sigma }_{01}& \dots \\ {\sigma }_{01}& {\sigma }_{11}& \dots \\ \vdots & \vdots & \ddots \end{array}\right)\right]\end{array}$$

We refer to this as the “linear trajectories, free covariance” model. Since this is a marginal model, rather than one that is conditional on participant, there are now no random effects for intercept and slope. Instead, an unstructured covariance matrix is used to allow correlation between measurements taken on the same person. An unstructured covariance matrix allows these correlations to take any value, as opposed to restricting to the specific functional form implied by the random slopes model.

A less restrictive model allows the mean trajectory in the control group to vary freely, while still requiring the treatment effect to be proportional to time:

7 $$\begin{array}{c}{y}_{ij}={\beta }_{j}+\gamma {z}_{i}{t}_{j}+{\epsilon }_{ij}\end{array}$$

where ${\beta }_{j}$ is the mean level of the outcome in the control group at time point $j$, and the residual error terms ${\epsilon }_{ij}$ are again distributed multivariate normally with an unstructured covariance matrix as in (6). We refer to this model as the “free control-group trajectory, free covariance” model. This model may be of use when there are non-linear trajectories, but the treatment effect between the groups is still linear.

Another, even less restrictive, model allows the trajectories in each group to vary freely over time, but still constrains the expected value of the outcome at baseline to be the same in the two groups:

8 $$\begin{array}{c}{y}_{ij}={\beta }_{j}+{\gamma }_{j}{z}_{i}+{\epsilon }_{ij}\end{array}$$

where ${\gamma }_{0}=0$, i.e. there is no treatment effect at baseline, and the residual error terms ${\epsilon }_{ij}$ are distributed multivariate normally with an unstructured covariance matrix, as in (6). We refer to this as the “free trajectories, free covariance” model. Again, the constraint that ${\gamma }_{0}=0$ takes advantage of the fact that the trial is randomised, and therefore that the two groups are on average the same at baseline, resulting in a more efficient analysis compared to allowing a separate expected value for each group at baseline [14].

Unlike the treatment effects in (1), (6) and (7), which all assume the treatment effect takes the form $\gamma {z}_{i}{t}_{j}$, the treatment effects in the free trajectories, free covariance model cannot be defined in such a way, as there is a separate treatment effect at each time point. As discussed further in the "Estimand of interest" section, for (8) we consider the difference in mean levels between the two treatment groups at the final time point of the trial, ${\gamma }_{{n}_{t}}$.

## Simulation study

The primary aim of the simulation study was to assess whether the empirical power of testing the treatment effect in the random slopes model in (1) differed from the power specified for the original sample size calculation when mean trajectories and/or treatment effects were non-linear. The secondary aim was to assess the performance of the less restrictive analysis models described in the "Possible alternative models for analysis" section when used at the trial analysis stage, having powered the trial using the random slopes model.

**Fig. 1:**
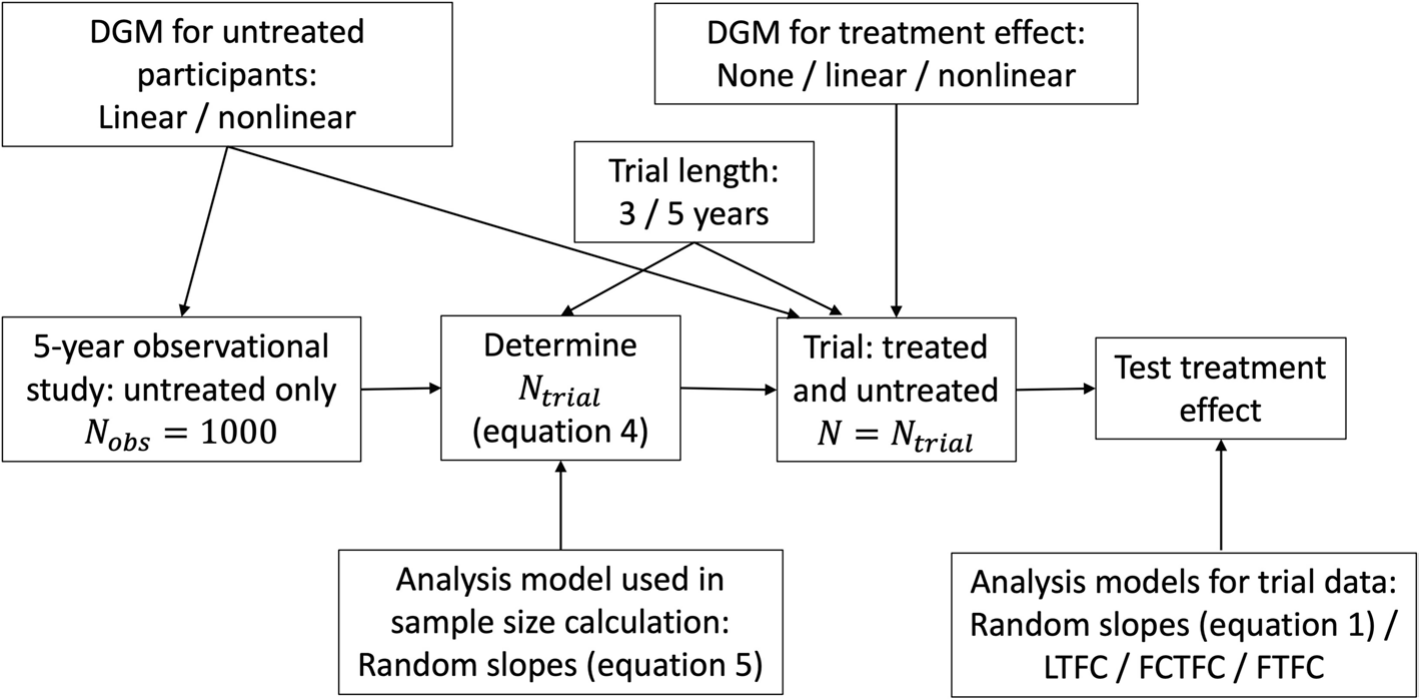
Graphical summary of the structure of the simulation. DGM: data generating mechanism. LTFC: linear trajectories, free covariance model. FCTFC: free control-group trajectory, free covariance model. FTFC: free trajectories, free covariance model

The general format of our simulations is as follows (see also graphical summary in Fig. 1):

1. Simulate previously collected data. Simulate an observational study of size ${N}_{obs}=1000$, using a particular mean trajectory (linear or non-linear; see the "Trajectories over time in people not receiving the trial treatment" section for details) plus random slopes and intercepts, and residual error.
2. Analyse previously collected data. Use the model in (5) to get estimates of ${\sigma }_{u0}^{2},{\sigma }_{u01},{\sigma }_{u1}^{2}$ and ${\sigma }_{e}^{2}$.
3. Calculate sample size. Calculate a sample size, ${N}_{trial}$, using (4), the estimates of the (co)variances obtained from analysing the simulated previously collected data in step 2, and pre-specified values of the target treatment effect, the number and timing of follow-up visits, and the Type I and Type II errors (see the "Sample size calculation" section).
4. Simulate trial data. Simulate a trial of size ${N}_{trial}$ calculated in step 3, with the same mean trajectory as used in step 1 and one of three possible forms for the treatment effect (see the "Treatment effect in the trial" section).
5. Analyse trial data (see the "Methods of analysis for the trial" section). The treatment effect at the final time point is saved, along with its SE.
6. Repeat 5000 times. The empirical power (along with other metrics; see the "Performance measures" section) is then calculated from the saved treatment effects and their SEs. 5000 repeats gives a Monte Carlo SE of $\sqrt{0.8\times 0.2/5000}=0.6\%$ for 80% power. Empirical powers of between 78.8% and 81.2% therefore have a 95% Monte Carlo confidence interval that covers a true power of 80%. The Monte Carlo SE for 5% Type I error is $\sqrt{0.05\times 0.95/5000}=0.3\%$, so observed Type I errors of between 4.4% and 5.6% have a 95% Monte Carlo confidence interval that covers a true Type I error of 5%.

In the following sections, we describe the elements of the simulation study in more detail.

Our simulations are loosely based on Expanded Disability Status Scale (EDSS) data from the MS-STAT trial [17], a phase 2 trial of simvastatin versus placebo in 140 patients with secondary progressive multiple sclerosis. The primary outcome was the annualised rate of whole-brain atrophy. EDSS was a secondary clinical outcome, and was collected at baseline, 12 months, and 24 months.

All simulations were run in Stata 14 [18]. Example code for generating and analysing the simulated data is given in section A9 of the Online Appendix. The reporting of this simulation study is based on the aims, data-generating mechanisms, estimands, methods, and performance measures structure suggested in [19].

### Trajectories over time in people not receiving the trial treatment

Trajectories for the ${i}^{th}$ person over integer-valued times ${t}_{j}$ from 0 to 5 years were simulated using the following model:

$${y}_{ij}=6+0.2f\left({t}_{j}\right)+{u}_{0i}+{u}_{1i}{t}_{j}+{e}_{ij}$$

where higher values indicate a worse outcome and the function $f\left({t}_{j}\right)$ determines whether the mean trajectory is linear or non-linear as follows:

- “Steady decline”: $f\left({t}_{j}\right)={t}_{j}$ (i.e. a linear trajectory)
- “Early decline”:$$f\left({t}_{j}\right)=-5\mathrm{exp}\left(-2{t}_{j}\right)+5$$
- “Late decline”: $$f\left({t}_{j}\right)=\frac{1}{4400}\mathrm{exp}\left(2{t}_{j}\right)$$
- “Intermediate decline”:$$f\left({t}_{j}\right)=\frac{-5}{1+\mathrm{exp}\left(-3\left(2.5-{t}_{j}\right)\right)}+5$$

Note that the non-linearity enters only in the fixed part of the model – the time component of the random effects, ${u}_{1i}{t}_{j}$, remains linear. This allows us to compare the different mean trajectories without also changing the variance structure at the same time.

**Fig. 2:**
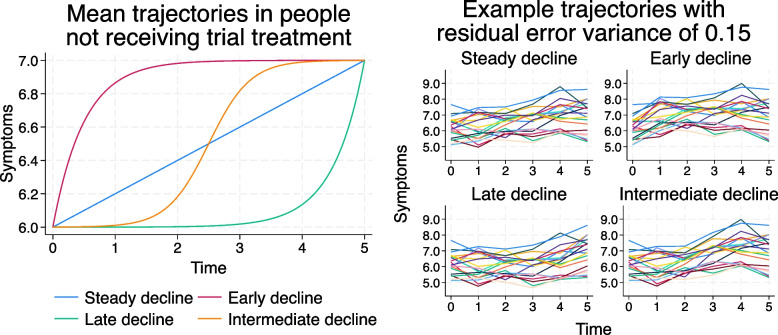
Mean trajectories over time in participants not receiving the trial treatment (left-hand panel); spaghetti plots for 20 people not on the trial treatment, with a residual error of ${\sigma }_{e}^{2}=0.15$ (right-hand panel)

Values of parameters were chosen such that all mean trajectories start from 6 at ${t}_{j}=0$ and end at 7 at ${t}_{j}=5$ (to 2 decimal places). The mean trajectories are shown in the left-hand panel of Fig. 2.

Parameter values used for the (co-)variances are: ${\sigma }_{u0}^{2}=0.5, {\sigma }_{u1}^{2}=0.01, {\sigma }_{u01}=0.5\times \sqrt{{\sigma }_{u0}^{2}{\sigma }_{u1}^{2}}$, ${\sigma }_{e}^{2}=0.15$. Spaghetti plots for 20 people not on the trial treatment are shown in the right-hand panel of Fig. 2. In a second set of simulations, the residual error variance was increased to ${\sigma }_{e}^{2}=2$.

In a second version of the intermediate decline trajectories, a person-level effect was added to the function for time so that the time of the steep decay of the trajectory occurs at different times for each person. Details are given in section A1 of the Online Appendix.

### Observational study and trial designs

The observational study in all scenarios was simulated to be 5 years long with a baseline visit and annual follow-up visits.

The trials were simulated to randomise participants in a 1:1 ratio to treatment or control. After a baseline visit, follow-up was by annual visits for either 5 years or 3 years, depending on the simulation scenario. All 5 years of the observational study were used to estimate the parameters for the sample size calculation, regardless of whether the proposed trial was 5 years or 3 years in length.

In addition, the average variances and co-variances from the simulated observational studies from certain scenarios were used to consider samples sizes calculated for 2, 4, 6 and 7-year trials with annual visits.

### Estimand of interest

If trajectories are truly linear, then the estimand of interest is the difference in slopes between the treatment groups. However, if trajectories are non-linear, then various alternative differences between treatment groups could be chosen as the estimand of interest. We could choose the difference in a linear contrast of the mean levels at the various follow-up times, or a difference in mean levels at a particular point in time. We think that a comparison of means at the end of follow-up is likely to have most appeal for researchers. We therefore consider the final time-point treatment effect to be an additional estimand of interest, i.e. the difference between the mean levels in the treated and untreated groups at 5 years for a 5-year trial and 3 years for a 3-year trial. In order to compare estimated treatment effects from the different models, when summarising our results, we convert all differences in slopes to the corresponding difference in mean levels at the end of follow-up. For the random slopes model with means at baseline in the two treatment groups constrained to be the same, we can switch from a difference in slopes to a difference in final levels by multiplying by the follow-up time.

### Treatment effect in the trial

Two different treatment effects were considered: one that is proportional to time:

$${y}_{ij}=6+0.2f\left({t}_{j}\right)+\gamma {z}_{i}{t}_{j}+{u}_{0i}+{u}_{1i}{t}_{j}+{e}_{ij}$$

and one that is proportional to the transformed time, $f\left({t}_{j}\right)$:

$${y}_{ij}=6+0.2f\left({t}_{j}\right)+\gamma {z}_{i}f\left({t}_{j}\right)+{u}_{0i}+{u}_{1i}{t}_{j}+{e}_{ij}$$

Since those in the control group arm have a mean change from baseline of $0.2f\left({t}_{j}\right)$, and the effect of additionally receiving treatment here is $\gamma f\left({t}_{j}\right)$, we refer to this treatment effect as “proportional to control group arm change”. For scenarios with ${\sigma }_{e}^{2}=0.15$, we set $\gamma$ to be $-0.05$, whilst for scenarios with ${\sigma }_{e}^{2}=2$ we took $\gamma$ to be $-0.1$.

For the steady decline trajectory, a treatment effect that is proportional to control group arm change would be the same as a treatment effect that is proportional to time. We therefore look at a different pattern of treatment effect that still has a steady decline trajectory in both groups: a treatment effect that delays deterioration (“delayed decline”). In the delayed decline scenario, the mean trajectory in the treated group shows no change for 1.25 units of time (2.5 units of time for scenarios with ${\sigma }_{e}^{2}=2$) and then mirrors the mean trajectory in the control group.

Parameters were chosen such that the expected treatment effect on the mean level at 5 years is -0.25 (to 2 decimal places) for all trajectories (-0.5 with ${\sigma }_{e}^{2}=2$). Therefore, all scenarios have the same distribution of outcomes at baseline and at 5 years (within values of ${\sigma }_{e}^{2}$), and hence the same treatment effect on the mean levels at 5 years. Some scenarios involve a 3-year trial, and the expected treatment effects at 3 years vary across trajectories when using an effect that is not proportional to time (see section A2, Online Appendix).

**Fig. 3:**
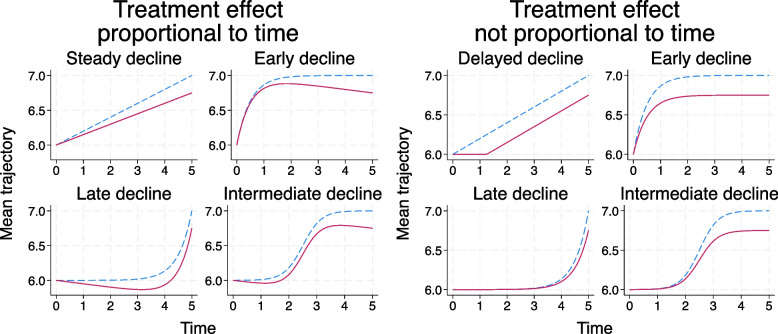
Plots of the mean trajectory in each treatment group of the trial for scenarios with residual error variance ${\sigma }_{e}^{2}=0.15$ (upper dashed line in each plot is for the control group and lower solid line for the treated group); treatment effects that are proportional to time are shown on the left and treatment effects that are not proportional to time on the right

The mean trajectories in the treated and control groups of the trial are shown in Fig. 3 for each of the treatment effects and with ${\sigma }_{e}^{2}=0.15$. The proportional treatment effects for the non-linear trajectories are not necessarily clinically plausible in all settings since the mean of the treated group improves during part of the follow-up time (as opposed to worsening but at a reduced rate compared to the control group). However, we included these scenarios so that we could examine the effect of a non-linear trajectory over time separately to a treatment effect that is not proportional to time.

Scenarios with no treatment effect were also considered, in order to assess the Type I error.

### Sample size calculation

Sample sizes for the trial, ${N}_{trial}$, are obtained from Eq. (4). Estimates of ${\sigma }_{u0}^{2}, {\sigma }_{u1}^{2}, {\sigma }_{u01}$ and ${\sigma }_{e}^{2}$ are obtained from fitting the random slopes model in Eq. (5) to the observational study. A Type I error of 5% and a power of 80% are used for all scenarios. The target treatment effect, $d$, is set to $-0.05$/year (corresponding to a difference in means of -0.25 at 5-years and -0.15 at 3 years) in scenarios with ${\sigma }_{e}^{2}=0.15$ and $-0.1$/year (-0.5 difference in means at 5-years and -0.3 for 3 years) in scenarios with ${\sigma }_{e}^{2}=2$, regardless of the type of treatment effect generated in the trial.

Note that these are the correct values of $d$ to use when simulating a proportional treatment effect in the trial. When a non-proportional treatment effect is used, the target treatment effect is no longer correct. We chose all mean trajectories to maintain a difference between the group means of -0.25 (or -0.5) at 5 years. At first glance, therefore, one might think that $-0.05$ (or $-0.1$) /year is still an appropriate value of $d$ to use for a 5-year trial with a non-proportional treatment effect. However, due to the way the fixed effect $\gamma$ from Eq. (1) is estimated, this is in fact not the case. Bamia et al. [20] show that when the model in (1) is fitted to data the estimate of $\gamma$ is a linear combination of the differences in the group means of the outcome at each time-point. The estimate of the trial treatment effect from the random slopes model is therefore dependent on the differences between the mean trajectories in the treated and control groups at the intermediate time points, and hence the estimate of $\widehat{\gamma }$ from model (1) is no longer equal in expectation to $d$ when a non-proportional treatment effect is simulated. The change in treatment effect is not predictable since it also depends on the estimated covariance structure. It can therefore become smaller or larger than $d$, depending on the data in question.

The same 5000 sample sizes for 5-year trials obtained from steps 1 and 2 above are used for each of the different treatment effect options, to minimise the computation time required for the simulations, and similarly for the 5000 3-year trial sample sizes. The distribution of sample sizes is therefore the same for all treatment effects, within each mean trajectory, for each of the trial lengths.

### Simulation scenarios

The following parameters were varied, in a factorial manner unless otherwise stated:

- Mean trajectory in those not receiving trial treatment: steady, early, late, intermediate decline (with and without extra random effect; intermediate decline with extra random effect only used with the smaller residual error variance below)
- Treatment effect: no treatment effect, proportional to time, proportional to control group arm change/delayed decline
- Length of trial: same length as observational study (both 5 years), different length from observational study (3-year trial)
- Residual error variance: ${\sigma }_{e}^{2}=0.15$, ${\sigma }_{e}^{2}=2$

### Methods of analysis for the trial

In step 5 above, the trial is analysed using the random slopes model defined in (1) and (2). Stata’s mixed command was used, with a pre-specified ordering of algorithms that were worked through until the model converges (see section A3, Online Appendix). The linear trajectories, free covariance model (Eq. (6)), free control-group trajectory, free covariance model (Eq. (7)), and the free trajectories, free covariance model (Eq. (8)) were also used. All estimation used Kenward-Roger small sample corrections [21].

### Performance measures

The following performance measures were calculated for each scenario:

- Convergence of model: how often the analysis models converged (both at the observational study and trial stage). Other performance measures are calculated for the simulated trials for which all models converged. We also recorded whether the estimated correlation between the random intercept and slopes was close to the boundary (magnitude $>0.99$) for the random slopes model.
- Power (when a treatment effect is present) or Type I error (for no treatment effect)
- Mean treatment effect
- Empirical SE (the standard deviation of the treatment effects across the simulated trials)
- Mean model-based SE (the square-root of the mean of the treatment effect variances from the simulated trials)
- Percentage bias in model-based SEs (as compared to the empirical SE)

## Results

Full tables and figures of results for all scenarios are given in the online appendix. We summarise the results in the following sections. Results are summarised for trials for which all models converged.

### Convergence

Convergence rates for the random slopes model were generally high (range: 99.1% to 100%). However, for certain mean trajectories, a large proportion of datasets analysed using the random slopes model gave estimates of the correlation between the random intercepts and slopes that are very close to the boundaries. This is not automatically flagged by mixed but could still indicate some issues with convergence. For example, out of the 4991 observational studies with an early decline trajectory in which the random slopes model converged, 3796 (76%) had a correlation of > 0.99 (Table A4.1, Online Appendix).

For the 5-year trials with lower residual variance, the early and late decline trajectories have particularly high percentages of correlations of magnitude > 0.99 (32% to 67%; Table A4.2, Online Appendix), whereas for the 3-year trials, the early and intermediate decline trajectories both have high percentages (80% to 100%; Table A4.2, Online Appendix). This reflects the large amounts of non-linearity added in these scenarios.

For the scenarios with ${\sigma }_{e}^{2}=2$, there were substantial numbers of datasets with correlations at the boundaries for all mean trajectories, even the steady decline ones (Tables A4.3 and A4.4, Online Appendix). For the steady decline trajectories, this effect can clearly not be attributed to non-linearity in the mean. Instead, the large residual variance is leading to highly variable datasets, some of which happen to no longer fit the random slopes model with a correlation that is bounded within -1 and + 1.

In practice, an analyst is likely to notice if the model estimate has ended on a boundary when analysing the observational study and explore alternative models. As a sensitivity analysis, we therefore summarised our results from the lower residual error scenarios for the early and late decline trajectories for those observational studies which gave a correlation of magnitude $\le$ 0.99. We found that patterns were broadly similar compared to including all simulations, results for which are given in the next sections. Full details can be found in the online appendix (section A6).

For some scenarios, the convergence rate using only the default Newton Raphson algorithm was low, and the use of alternative algorithms improved the convergence rate (see section A4, Online Appendix). For example, for a 3-year trial with no treatment effect, smaller residual error variance, and the early decline trajectory only 3526 random slopes models converged using the Newton Raphson algorithm. Use of alternative algorithms and options led to a further 1443 converged models. In these models, the correlations between the random intercepts and slopes were found to have estimates very close to the boundary.

### Sample sizes

**Fig. 4:**
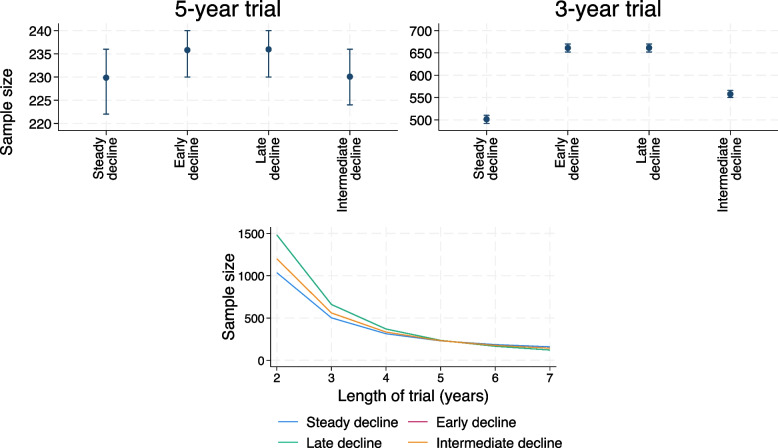
Top panels: mean sample sizes and inter-quartile range, as determined using the observational studies with smaller residual error variance; 5-year (left) and 3-year (right) trial. Bottom panel: sample sizes determined using mean covariance parameters estimated in the 5000 observational studies (or the true data generation parameters for steady decline trajectory) for a range of trial lengths. Note that the lines for late and early decline lie directly on top of each other

**Fig. 5:**
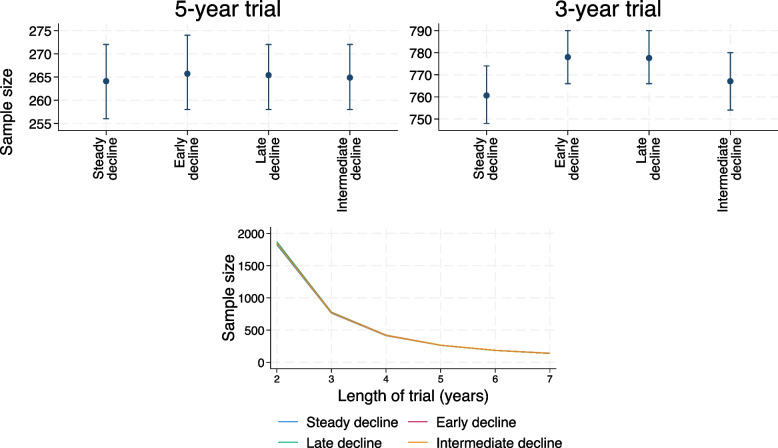
Top panels: mean sample sizes and inter-quartile range, for a 5-year (left) and 3-year trial (right) calculated using the observational studies, for the larger residual error variance. Bottom panel: sample sizes determined using mean covariance parameters estimated in the 5000 observational studies (or the true data generation parameters for steady decline trajectory) for a range of trial lengths

The sample sizes calculated from the observational studies for the 5-year and 3-year trials are given in Fig. 4 for the smaller residual error variance, and in Fig. 5 for the larger residual error variance.

Figure 4 shows that for a 5-year trial (top left-hand panel), the sample sizes for the non-linear trajectories are similar to (but a little higher than) those for the steady decline trajectory. For the 3-year trial (top right-hand panel), however, the sample sizes for the non-linear trajectories are considerably higher than for the steady decline trajectory.

The bottom panel of Fig. 4 shows the sample sizes that would be specified for a range of trial lengths from 2 to 7 years, all with annual visits. The true data generation parameters were used to give the sample sizes for the steady decline trajectory, and the mean estimated (co)variance values from the observational studies for the non-linear trajectories. We see that for the smaller residual error variance, the non-linear trajectories’ sample sizes are larger than the linear trajectory sample size for trials that are shorter than the observational study, and smaller for longer trials. The differences between the sample sizes increase as the difference in length between the trial and the observational study increases.

Figure 5 shows that the differences between the linear and non-linear trajectory sample sizes are much smaller for the scenarios with larger residual error variance.

### Free control-group trajectory, free covariance model

We found that for the scenario with steady decline trajectories, a proportional treatment effect, a 5-year trial and a smaller residual error variance, the free control-group trajectory, free covariance model gave exactly the same treatment effects as the linear trajectory, free covariance model, with very similar SEs (Table A6.2.1, Online Appendix). The differences between the SEs were small enough that the power was the same to 1 decimal place. We therefore excluded the free control-group trajectory, free covariance model as an analysis model in the other scenarios.

### Smaller residual error variance, ${\upsigma }_{\mathrm{e}}^{2}=0.15$

In this section we report the results for the scenarios with the smaller residual error variance, for each of the three possible types of treatment effect.

#### No treatment effect

Here we summarise the scenarios with no treatment effect in the trial, to check the Type I error rates of our analysis models before going on to estimate their power.

**Fig. 6:**
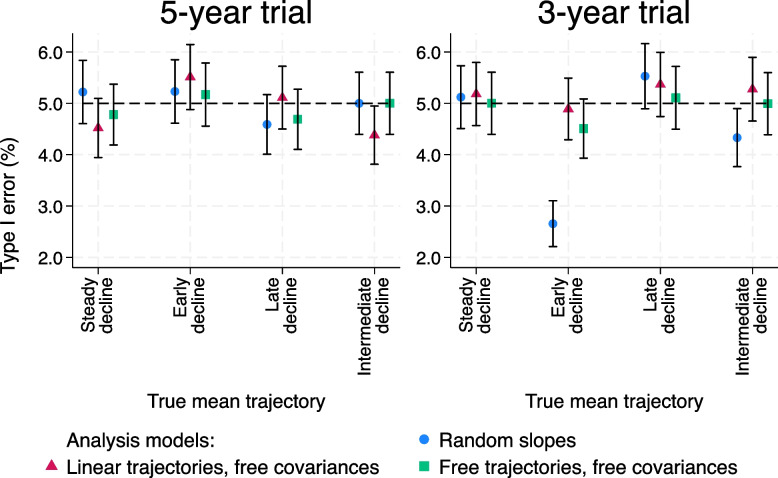
Type I errors for trials with no treatment effect and smaller residual error variance. Left-hand panel: 5-year trial. Right-hand panel: 3-year trial. Ranges plotted are $\pm 1.96$ times the Monte Carlo standard error

For a 5-year trial estimated treatment effects are close to zero (Table A6.1.1, Figure A6.1.1; Online Appendix), and Type I errors (Fig. 6, left-hand panel) are generally close to 5%, as expected.

For a 3-year trial, the mean treatment effects are still close to zero (Table A6.1.3, Figure A6.1.3; Online Appendix). However, Type I errors are conservative for the random slopes model for some mean trajectories. The Type I error is about half the nominal level for the early decline trajectory, and slightly below 5% for the intermediate decline trajectory (Fig. 6, right-hand panel). This is driven by upward bias in the random slopes model-based SE compared to its empirical SE for those mean trajectories, a feature that remains in the 3-year trial scenarios when a treatment effect is added (Tables A6.2.3 and A6.3.3, Online Appendix).

The incorrect Type I errors for these mean trajectories reflect the large amounts of non-linearity added, especially when only looking at 3 years of follow-up (see Fig. 2, from $t=0$ to $t=3$). The mean trajectories are extreme enough that the random slopes model is no longer fitting well.

#### Treatment effect proportional to time

We now begin to address our primary aim: to estimate the power of the random slopes model, in the first instance when there is a proportional treatment effect in the trial. We also look at the performance of our other two analysis models for our secondary aim.

**Fig. 7:**
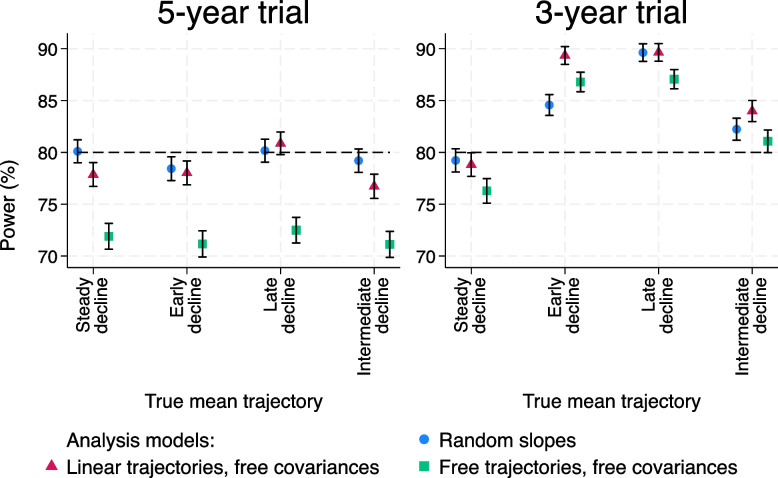
Powers for trials with a proportional treatment effect and smaller residual error variance. Left-hand panel: 5-year trial. Right-hand panel: 3-year trial. Ranges plotted are $\pm 1.96$ times the Monte Carlo standard error

Firstly, the empirical power for the steady decline trajectory and random slopes model is indeed close to the 80% power used in the sample size calculation, for both a 5-year and a 3-year trial (Fig. 7). The two less restrictive models have lower power when the assumptions of the random slopes model hold, as expected. The power loss for the free trajectories, free covariance model is much greater than for the linear trajectories, free covariance model.

For a 5-year trial, all models and mean trajectories have treatment effects close to that expected (Figure A6.2.1, Online Appendix). Power is close to 80% for the random slopes model for all mean trajectories (Fig. 7; left-hand panel) – having a non-linear trajectory does not seem to have a large effect on the power for a 5-year trial. The free trajectories, free covariance model loses power in all scenarios, as expected since the trial was powered for the more efficient random slopes model. While the treatment effect and its SE were estimated without bias by the free trajectories, free covariance model, the SE is larger than that for the random slopes model and so power is lost.

For a 3-year trial (right-hand panel of Fig. 7), the story is more complex. The powers for the random slopes model for the non-linear trajectories are generally considerably greater than the nominal 80% used in the sample size calculation (even for the early decline trajectory which has a low Type I error as seen in Fig. 6). This is not due to an incorrectly estimated treatment effect, since the mean treatment effects are again close to those expected (Figure A6.2.3, Online Appendix).

Some insight into this result can be gained by looking at the sample sizes specified by the observational study for the 5-year and 3-year trials (Fig. 4). The sample sizes for the non-linear trajectories are very similar to those for the steady decline trajectory for the 5-year trials, but are larger for the 3-year trial, leading to the over-powering seen above.

#### Treatment effect not proportional to time

We now assess the power of our analysis models in the presence of a treatment effect that is either proportional to control group arm change or delayed decline.

**Fig. 8:**
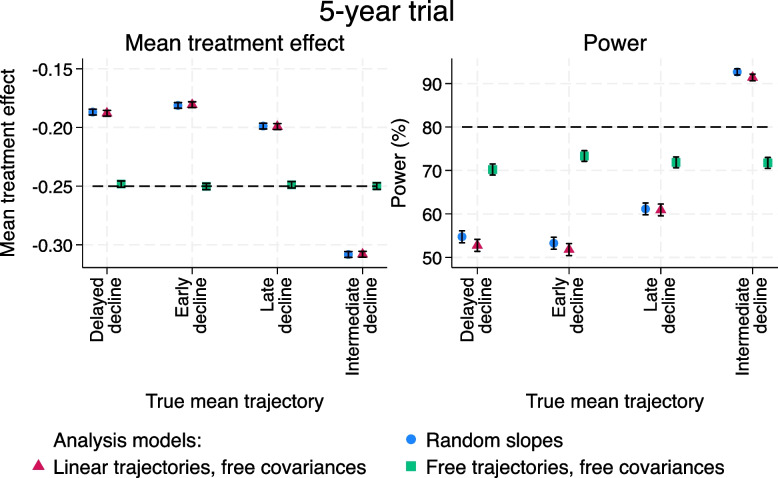
5-year trials with non-proportional treatment effects and smaller residual error variance. Left-hand panel: mean treatment effect at 5 years, converted from differences in slopes between the treatment groups for the random slopes and linear trajectories, free covariances models. Right-hand panel: power. Ranges are $\pm 1.96$ times the Monte Carlo standard error

For a 5-year trial, the random slopes model and the linear trajectories, free covariance model both have substantially different final time-point treatment effects (as converted from a difference in slopes between the treatment groups) to those expected (Fig. 8; left-hand panel). The powers are therefore far from nominal (Fig. 8; right-hand panel). Using the linear trajectories, free covariance model gives very similar results to random slopes. It is necessary to use the free trajectories, free covariance model to get the correct mean treatment effect. But using such an analysis model when the sample size has been calculated assuming a random slopes model leads to loss of power compared to the nominal level of 80% just as in Fig. 7 (range: 70.2 to 73.3%). It does maintain substantially more power than the other analysis methods for 3 out of 4 of the mean trajectories, however. For the remaining mean trajectory (intermediate decline), the magnitude of the treatment effect is considerably over-estimated by the random slopes and linear trajectories, free covariance models, leading to a power of over 90%.

**Fig. 9:**
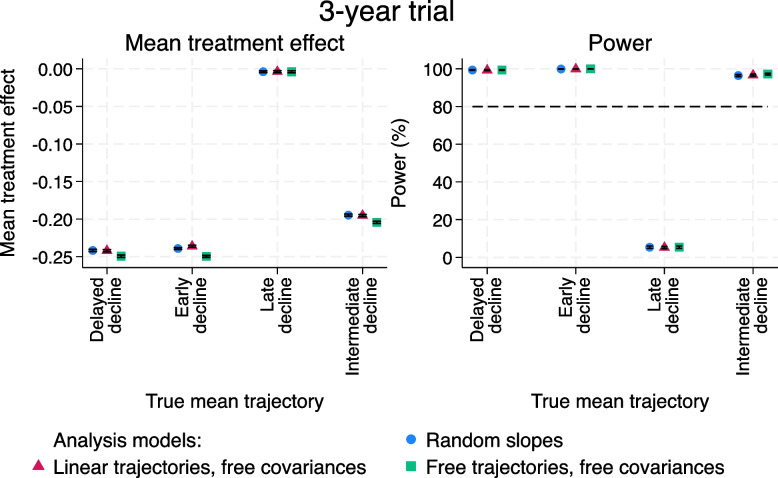
3-year trials with non-proportional treatment effects and smaller residual error variance. Left-hand panel: mean treatment effect at 3 years, converted from differences in slopes between the treatment groups for the random slopes and linear trajectories, free covariances models. Right-hand panel: power. Ranges are $\pm 1.96$ times the Monte Carlo standard error

These issues are exacerbated when the length of the trial is 3 years (Fig. 9). In this set of scenarios, the true treatment effect now differs between the mean trajectories (see Table A2.1, Online Appendix). For example, there is hardly any difference between the two treatment groups at 3 years for the late decline trajectory as the separation between the groups occurs after this point. This leads to very variable powers (Fig. 9; right-hand panel), depending on whether the true treatment effect is much larger or much smaller than -0.15 at 3 years.

### Larger residual error variance, ${\upsigma }_{\mathrm{e}}^{2}=2$

The patterns of results for the scenarios with a larger residual error variance were often similar to those for the smaller residual variance. The Type I error for the random slopes model for an early decline trajectory and a 3-year trial was no longer conservative (Table A7.1.2, Online Appendix).

For a proportional treatment effect, the power of the random slopes model was close to 80% for all mean trajectories for both 3- and 5-year trials (Figures A7.2.1 and A7.2.2, Online Appendix). This is unlike the equivalent scenarios with the smaller residual error variance, where the power for the random slopes model moved away from nominal levels for the 3-year trial with non-linear trajectories.

Again, some insight into this result can be gained by looking at the sample sizes specified by the observational studies. The sample sizes specified for a 3-year trial and non-linear trajectories are closer to those for the steady decline trajectory for the larger residual variance scenarios than in the corresponding scenarios with a smaller residual variance (Fig. 5; right-hand panel).

When using a non-proportional treatment effect with the larger residual variance, the power is again not at nominal levels for all mean trajectories and analysis models for a 5-year trial. However, for all mean trajectories except late decline the treatment effect for the random slopes model is over-estimated, leading to greater than nominal power levels (Figure A7.3.1, Online Appendix). Results for the 3-year trial with a non-proportional treatment effect showed similar patterns to the scenarios with lower residual variance (Figure A7.3.2, Online Appendix).

### Intermediate decline with extra random effect

For the scenarios with no treatment effect, the Type I errors were close to 5% (Tables A6.1.1 and A6.1.3, Online Appendix). For the proportional treatment effect, power was close to nominal for a 5-year trial, with some loss for the 3-year trial (Figure A5.1, Online Appendix).

There were some differences in the mean treatment effect from the target treatment effect when using the random slopes model in the scenarios with a treatment effect proportional to control group arm change, although somewhat less than for the other non-linear trajectories (Tables A6.3.1 and A6.3.3, Online Appendix). This leads to inflated powers (Figure A5.1, Online Appendix), but ones that are not too far from the nominal 80%.

## Discussion

We have summarised our results in terms of any impact on power in Table 1.

**Table 1:** Summary of results by analysis model, trajectory type, treatment effect type and whether proposed trial is the same length as the observational study

Analysis model	Linear or non-linear trajectory in DGM	Proportional or non-proportional treatment effect in DGM	Observational study length relative to trial length	Impact on power	Notes
Random slopes model	Linear	Proportional	Same	None noticeable	Assumptions of sample size calculation are met
			Different	None noticeable	
	Non-linear	Proportional	Same	None noticeable	
			Different	Can differ from nominal	Close to nominal when residual error is large relative to between-person variance, but can differ from nominal when residual error is small. Power can be higher or lower than nominal
	Non-linear	Non-proportional	Same	Can be very far from nominal	Differing power is partially due to incorrect target treatment effect used in sample size calculation. Power can be higher or lower than nominal
			Different	Can be very far from nominal	
Free trajectories, free covariance^a^	Linear	Proportional	Same	Power loss	Power loss is greater when residual error is large relative to between-person variance
			Different	Power loss	
	Non-linear	Proportional	Same	Power loss	Power loss is greater when within-person error is large relative to between-person error
			Different	Can differ from nominal	Power can be higher or lower than nominal
	Non-linear	Non-proportional	Same	Some power loss	
			Different	Can be far from nominal	Differing power is partially due to incorrect target treatment effect used in sample size calculation. Power can be higher or lower than nominal

^a^Sample size for the free trajectories, free covariance model is calculated under the assumption of a random slopes model

*DGM *Data generating mechanism

### Impact of wrongly assuming a linear trajectory, when the treatment effect is truly proportional to time

The empirical power for the random slopes model was close to nominal when the observational data and the planned trial had the same duration and visit schedule, regardless of whether the mean trajectories are linear or not, provided that the treatment effect is correctly specified.

This result agrees with the simulation study in reference [5], in which Hu et al. found that the sample size calculation from a random slopes model was relatively robust to the addition of a quadratic term to the mean trajectory in both the control and the treatment group (such that the treatment effect was still proportional to time). Their simulation study looked only at the data generation mechanism for the trial, and not at any previously collected data used to provide estimates of variance components, so our finding builds on theirs.

Using an observational study with a different schedule of visits to that in the proposed trial can impact power when there is non-linearity, even for a proportional treatment effect. When using a 5-year observational study to specify sample sizes for a 3-year trial, empirical powers for the non-linear trajectories are higher than nominal. If we had used a 3-year observational study to specify the sample sizes for a 3-year trial, we might have again seen that a non-linear mean trajectory has little effect on the empirical power.

To understand why powers are high for shorter trials in the presence of non-linearity, we first consider the effect of shortening trials when trajectories are linear. When mean trajectories are truly linear, the change in sample size for a shorter trial is governed by the size of the residual variance relative to the between person variance. When the residual variance is larger, the effect of shortening follow-up is greater than for a smaller residual variance. Intuitively, there is a greater gain in efficiency (in terms of sample size reduction) from increasing the trial’s length when the residual within-person error is greater. When mean trajectories are non-linear, the residual error in the random slopes model is over-estimated whereas the intercept and slope variances are generally under-estimated (Figures A8.1.2 and A8.1.3, Online Appendix). The ratio of the residual error to the between person variance is therefore over-estimated in the presence of non-linearity, and the sample size for a shorter trial is therefore over-estimated compared to the adjustment that would be required if mean trajectories were linear. These larger sample sizes are then leading to over-powering in the 3-year trials.

The incorrect powering for a 3-year trial is less of an issue for a greater residual error variance, since the large residual error variance is dominating the sample size calculation, and the residual variance is generally over-estimated by only a small amount for the non-linear trajectories (less than 5% over-estimated, compared to up to around 60% overestimated for the lower residual error variance).

While we have only looked at a 3-year trial versus a 5-year observational study in our simulation study, these findings are likely to apply to any trial with a different length or pattern of visits to the observational study. For example, the bottom panel of Fig. 4 shows that sample sizes specified by a 5-year observational study for different length trials differ for non-linear compared to linear trajectories. This is likely to correspond to empirical powers that differ from nominal levels for these non-linear trajectories, as for the 3-year trial. For the parameters we considered, sample sizes are overestimated for trials shorter than the observational study and underestimated for longer trials. However, if not restricting to annual visits, it is possible to design a shorter trial with more frequent follow-up visits for which the sample size would be underestimated. For example, using a 5-year observational study with annual visits to specify the sample size for a 4.5-year trial with 6-monthly visits gives slightly smaller sample sizes for non-linear trajectories compared to linear.

### Impact of wrongly assuming the treatment effect is proportional to time

Having a non-proportional treatment effect does result in incorrectly powered trials, even for a trial that matches the visit schedule of the observational study. Bamia et al. [20] show that the treatment effect estimated from a random slopes model is a linear combination of the differences in means between the trial treatment arms at the follow-up times. This implies that even though the final time point treatment effect at 5 years is kept the same for our non-proportional treatment effect as in the other scenarios, the difference in slopes from the random slopes model is not. The altered means of the non-linear trajectories earlier in follow-up changes the expected treatment effect from the random slopes model, which can be larger or smaller than the target treatment effect used in the sample size calculation. Furthermore, the multipliers in the linear combination of mean differences depend on the estimated variance components. The estimated treatment effect from the random slopes model can therefore differ when, for example, the residual error variance is changed, even for the same mean trajectories. Our results echo Bamia et al.’s findings that the weights implicitly used by the random slopes model to estimate the treatment effect are not particularly intuitive and can lead to unexpected estimation of non-proportional treatment effects.

The incorrect powering is therefore in part due to the wrong target treatment effect being used in the sample size calculation. This leads to over- or under-powered trials, depending on the size of the estimated treatment effect relative to the target treatment effect. When the treatment effect estimated by the random slopes model is close to the target treatment effect, the effect on the power appears to be relatively small. For example, for the intermediate decline trajectory with extra random effect, the empirical power is only a little inflated for a 3-year trial with a non-proportional treatment effect, at 83%. The random slopes model estimates the treatment effect to be -0.055/year, only slightly larger in magnitude than the -0.05/year used in the sample size calculation. In contrast, the mean treatment effect for the random slopes model for the early decline trajectory is -0.080/year, leading to a power of almost 100%. Using an incorrect target treatment effect appears to have a much larger effect on the power than non-linearity of the mean trajectories in this case, although this may not hold more generally for other patterns of non-linearity.

### Impact of using other analysis models

As expected, using a less restrictive model to analyse the trial having powered for a random slopes model results in some loss of power when trajectories are truly linear. This is also often the case for the non-linear trajectories. For example, the free trajectories, free covariance model has powers of 70–75% for the scenarios with a smaller residual error variance, and the power loss is greater when the residual error variance is increased relative to the size of the between-person variance.

Analysts writing a statistical analysis plan for a future trial may be unsure whether any treatment effect will have a shape that is compatible with the random slopes model. In such a case, one option would be to use the random slopes model as a first-line approach, but to specify that the free trajectories, free covariance model will be used as a sensitivity analysis. The potential power loss induced by switching to a less constrained analysis model should be considered, perhaps by powering to a higher level with the random slopes model if that is possible.

If there is strong reason to suspect a non-linear treatment effect in the proposed trial, then the difference in slopes between the groups is not an appropriate estimand. Instead, interest may lie in the difference in means between the two groups at the end of follow-up, and a free trajectories, free covariance model could be used at both the sample size calculation and analysis stage. Alternatively, if a specific polynomial form for the treatment effect is suspected, then the trial could be planned on that basis [22, 23]. However, note that the models in these references do not constrain the treatment effect at baseline to be zero, and so will be inefficient in a randomised trial setting.

We also considered the linear trajectories, free covariance model. This model gives the same treatment effect as the free control-group trajectory, free covariance model. An analyst might therefore consider its use when there are non-linear trajectories, but the treatment effect is still expected to be linear or close to linear. However, we found that such a model does not gain much in terms of performance compared to the random slopes model.

Fitting a random slopes model can result in boundary problems, with an estimated correlation between random slopes and intercepts of + 1 or -1. The rate of convergence to a boundary was greater for mean trajectories with large amounts of non-linearity, and for scenarios with a larger residual error variance. Using a model with an unstructured covariance model avoids this particular convergence issue.

### Limitations and future work

Our simulation study considered a wide range of scenarios and mean trajectories. However, as always with a simulation study, these cover a small fraction of all possible choices. For example, we chose to only consider 80% power. At 90% power trial sample sizes will be larger, and we anticipate that this will provide more protection against violation of assumptions due to the central limit theorem.

In addition, we considered only one size of observational study. We anticipate that varying the size of the observational study is only likely to affect the results through introducing more (or less) uncertainty in the model estimates from the observational study (these then being used in the sample size calculation). We chose a large sample size of 1000 participants, in order that those estimates are relatively precise, but that the size of the dataset is still in line with datasets that may be available for trial planning. Using a smaller sample size for the observational study would result in larger Monte Carlo errors but not alter patterns of results. We chose to look at the impact of increasing the uncertainty by including scenarios with a larger residual error variance instead.

Under certain circumstances investigators may want to consider using the free trajectories, free covariance model in the sample size calculation. The methodology outlined in Frost et al. [4] could be used for this purpose. However, using the random slopes model allows us to interpolate (and even extrapolate) the estimated covariance structure in the observational study so that we can consider the sample sizes needed for trials with different lengths and patterns of visits. Since the covariance structure is unstructured in the free trajectories, free covariance model, we would lose the ability to look at patterns of visits that are not in the original observational data set and we would need data with exactly the time points of the proposed trial. To consider visit schedules not contained within the observational study, it would be necessary to impose a parametric structure for the covariance matrix, and further work would be required to assess how such an approach works in practice. In addition, further work could include exploring the potential benefits of considering a range of plausible non-proportional treatment effects when specifying the sample size.

### Software

We conducted our simulation study in Stata. While many of our results will be relevant for users of other software packages, there are some differences that could have an impact. For example, we found that for some datasets the intercept-slope correlation from the random slopes model was estimated on the boundary. Analysing a handful of such datasets using the lme command from the nlme package [24], and the lmer command from the lme4 package [25], in R leads to warning messages saying that the model has not converged. If we had used R instead of Stata, we might therefore have categorised many more datasets as being non-convergent than we did in Stata. Although if the non-converged estimates given by the R commands were used, they would likely give very similar results to ours.

If we had used SAS’s proc mixed [26] command with the default settings, we would probably have seen similar results as in Stata. However, in SAS, it is possible to change the settings such that the correlation can be estimated outside of the boundary, which might have yielded some differences to our results. This does leave the question of what a correlation of greater than 1 (say) means, however. In practice, if an analyst used SAS and observed a correlation that is outside the boundary, they may feel that a random slopes model is not an appropriate one for their data.

In Stata, we used a pre-specified ordering of algorithms and model options for the random slopes model. This increased the number of datasets for which the random slopes model converged, although the correlation between the random intercepts and slopes often converged on the boundary for these datasets. The use of these alternative algorithms could be of use to an analyst who is trying to determine why a model is not converging using the default settings.

## Conclusion

When the treatment effect in the trial is proportional to time, having non-linear trajectories has little or no effect on the empirical power, unless the proposed trial has a different visit schedule to the observational study. The extent of the effect on power depends on the underlying data generation parameters, for example the size of the residual error variance. If investigators anticipate that the treatment effect in the proposed trial will be proportional to time (or close to this), then powering the trial assuming a random slopes model should yield empirical powers close to those expected. However, if there are marked non-linearities in the trajectories, then observational data with the same visit schedule as the proposed trial should be used where possible, especially in settings where the random intercepts and slopes variances are comparable in size to the residual variance. Alternatively, investigators could switch the analysis model to one with a less restrictive structure, for example, the free trajectories, free covariance model, and power accordingly.

When treatment effects are not proportional to time, this has a large impact on the empirical power, even for a trial with the same visits as the observational study. If investigators want to guard against the possibility of a non-proportional treatment effect, a free trajectory, free covariance model could be specified as a second line analysis method, bearing in mind that power will be lost when using a less restrictive model if the linearity assumptions are in fact true. Alternatively, if investigators have strong reasons to suspect any treatment effect in the proposed trial will not be proportional to time, then such a model should be used at the sample size calculation stage.

### Supplementary Information

**Additional file 1.** Online appendix.

## Data Availability

The simulated datasets used and/or analysed during the current study are available from the corresponding author on reasonable request.

## Abbreviations

SE: Standard
DGM: Data generating mechanism
LTFC: Linear trajectories, free covariance model
FCTFC: Free control-group trajectory, free covariance model
FTFC: Free trajectories, free covariance model
EDSS: Expanded Disability Status Scale
