# Supplementary material for: How important is the linearity assumption in a sample size calculation for a randomised controlled trial where treatment is anticipated to affect a rate of change?
Source: BMC Med Res Methodol. 2023 Nov 21;23:274. doi: 10.1186/s12874-023-02093-2 (PMC10664473; doi:10.1186/s12874-023-02093-2)
Supplement: Supplementary file 1 — Additional file 1. Online appendix. [file 12874_2023_2093_MOESM1_ESM.docx]

## Online appendix

### A1. Intermediate decline trajectory with extra random effect

In a second version of the intermediate decline trajectories, a person-level effect $v_{0i}$ was added to the function for time: $f\left( t_{j} \right)=\frac{-5}{1+\exp\left( -3\left( 2.5+v_{0i}-t_{j} \right) \right)}+5$, so that the time of the steep decay of the trajectory occurs at different times for each person. This is likely to be more realistic than the entire cohort declining fastest at the same time, halfway through the follow-up period. The extra random effect was normally distributed with mean 0 and variance $\sigma_{v0}^{2}=1$.

Due to the extra random effect, the variability of this trajectory is greater than the other trajectories. It is therefore not directly comparable to the other trajectories. It also leads to some small differences in the mean levels and treatment effect. The mean level at baseline is 6.02 and the mean level in the control group at 5 years is 6.98.

For a residual error variance of 0.15, the treatment effect on the mean level that is proportional to time is still ‑0.25 at 5 years and ‑0.15 at 3 years. The non-proportional treatment effect (proportional to placebo arm change) is ‑0.246 at 5 years and ‑0.167 at 3 years.

### A2. Expected treatment effects in 3-year trial

Table A2.1: true mean differences between treatment groups at 3 years. Note that the true treatment effect is zero in scenarios with no treatment effect. r.e. = random effect

| Treatment effect | Trajectory | Placebo arm mean | Residual error $\sigma_{e}^{2}=0.15$ | | Residual error $\sigma_{e}^{2}=2$ | |
| --- | --- | --- | --- | --- | --- | --- |
|  |  |  | Treated arm mean | Treatment effect on mean | Treated arm mean | Treatment effect on mean |
| Proportional to time | Steady decline | 6.60 | 6.45 | -0.15 | 6.30 | -0.30 |
|  | Early decline | 7.00 | 6.85 | -0.15 | 6.70 | -0.30 |
|  | Late decline | 6.02 | 5.87 | -0.15 | 5.72 | -0.30 |
|  | Intermediate decline | 6.82 | 6.67 | -0.15 | 6.52 | -0.30 |
|  | Intermediate decline with extra r.e. | 6.67 | 6.52 | -0.15 | n/a | n/a |
| Not proportional to time | Delayed decline | 6.60 | 6.35 | -0.25 | 6.10 | -0.50 |
| Proportional to placebo arm change | Early decline | 7.00 | 6.75 | -0.25 | 6.50 | -0.50 |
|  | Late decline | 6.02 | 6.01 | -0.005 | 6.01 | -0.01 |
|  | Intermediate decline | 6.82 | 6.61 | -0.20 | 6.41 | -0.41 |
|  | Intermediate decline with extra r.e. | 6.67 | 6.50 | -0.17 | n/a | n/a |

### A3. Details of algorithms used with mixed

As described in Section 3.6 of the main paper, Stata’s mixed command was used to fit the random slopes model, with the default Newton-Raphson (NR) fitting algorithm in the first instance. If the model did not converge (i.e. it was classed by Stata as non-convergent), the model was re-run using the Davidon-Fletcher-Powell (DFP) algorithm (using the technique(dfp) option), and then the Broyden-Fletcher-Goldfarb-Shanno (BFGS) algorithm (technique(bfgs)) if still not convergent. If the model did not converge using the BFGS algorithm, the model was re-run with the matlog option, which parameterises variance components as logs during optimisation (rather than the default square root). If none of these options worked, the model was recorded as non-convergent.

### A4. Correlations between random intercepts and slopes

| Table A4.1: Mean correlation between random intercepts and slopes for random slopes model: observational studies | | | | | | | |
| --- | --- | --- | --- | --- | --- | --- | --- |
| Trajectory | Number converged | Mean correlation between intercepts and slopes | Number converged with correlation > 0.99 or  < -0.99 | Number converged using NR algorithm | Number converged using DFP algorithm (and correlation  > 0.99 or  < -0.99) | Number converged using BFGS algorithm (and correlation  > 0.99 or  < -0.99) | Number converged using matlog option (and correlation  > 0.99 or  < -0.99) |
| Steady decline | 5000 | 0.50 | 0 | 5000 | 0 (0) | 0 (0) | 0 (0) |
| Early decline | 4991 | 0.98 | 3796 | 4123 | 682 (682) | 155 (155) | 31 (31) |
| Late decline | 4993 | 0.98 | 3786 | 4178 | 627 (627) | 165 (165) | 23 (23) |
| Intermediate decline | 5000 | 0.64 | 0 | 5000 | 0 (0) | 0 (0) | 0 (0) |
| Intermediate decline (extra r.e.) | 5000 | 0.55 | 0 | 5000 | 0 (0) | 0 (0) | 0 (0) |
| NR = Newton-Raphson; DFP = Davidon-Fletcher-Powell; BFGS = Broyden-Fletcher-Goldfarb-Shanno; r.e. = random effect | | | | | | | |

| Table A4.2: Mean correlation between random intercepts and slopes for random slopes model: trials | | | | | | | | | |
| --- | --- | --- | --- | --- | --- | --- | --- | --- | --- |
| Trial length (years) | Treatment | Trajectory | Number converged | Mean correlation between intercepts and slopes | Number converged with correlation  > 0.99 or  < -0.99 | Number converged using NR algorithm | Number converged using DFP algorithm (and correlation  > 0.99 or  < -0.99) | Number converged using BFGS algorithm (and correlation  > 0.99 or  < -0.99) | Number converged using matlog option (and correlation  > 0.99 or  < -0.99) |
| 5 | none | Steady decline | 5000 | 0.51 | 0 | 5000 | 0 (0) | 0 (0) | 0 (0) |
|  |  | Early decline | 4990 | 0.95 | 3226 | 4309 | 564 (564) | 101 (101) | 16 (16) |
|  |  | Late decline | 4991 | 0.95 | 3308 | 4217 | 629 (629) | 123 (123) | 22 (22) |
|  |  | Intermediate decline | 5000 | 0.65 | 42 | 4992 | 7 (7) | 1 (1) | 0 (0) |
|  |  | Intermediate decline (extra r.e.) | 5000 | 0.56 | 7 | 5000 | 0 (0) | 0 (0) | 0 (0) |
|  | proportional | Steady decline | 5000 | 0.51 | 0 | 5000 | 0 (0) | 0 (0) | 0 (0) |
|  |  | Early decline | 4987 | 0.95 | 3263 | 4300 | 559 (559) | 106 (106) | 22 (22) |
|  |  | Late decline | 4991 | 0.95 | 3334 | 4178 | 670 (670) | 117 (117) | 26 (26) |
|  |  | Intermediate decline | 5000 | 0.66 | 63 | 4989 | 11 (11) | 0 (0) | 0 (0) |
|  |  | Intermediate decline (extra r.e.) | 5000 | 0.56 | 7 | 4999 | 1 (1) | 0 (0) | 0 (0) |
|  | non-proportional | Delayed decline | 5000 | 0.52 | 1 | 5000 | 0 (0) | 0 (0) | 0 (0) |
|  |  | Early decline | 4991 | 0.87 | 1613 | 4653 | 294 (294) | 35 (35) | 9 (9) |
|  |  | Late decline | 4991 | 0.87 | 1622 | 4676 | 271 (271) | 35 (35) | 9 (9) |
|  |  | Intermediate decline | 5000 | 0.62 | 13 | 4997 | 2 (2) | 1 (1) | 0 (0) |
|  |  | Intermediate decline (extra r.e.) | 5000 | 0.55 | 2 | 5000 | 0 (0) | 0 (0) | 0 (0) |
| 3 | none | Steady decline | 5000 | 0.52 | 75 | 4985 | 14 (14) | 1 (1) | 0 (0) |
|  |  | Early decline | 4969 | 1.00 | 4969 | 3526 | 1158 (1158) | 245 (245) | 40 (40) |
|  |  | Late decline | 4993 | 0.52 | 21 | 4990 | 2 (2) | 1 (1) | 0 (0) |
|  |  | Intermediate decline | 4987 | 0.99 | 4737 | 3641 | 1172 (1172) | 165 (165) | 9 (9) |
|  |  | Intermediate decline (extra r.e.) | 5000 | 0.43 | 0 | 5000 | 0 (0) | 0 (0) | 0 (0) |
|  | proportional | Steady decline | 5000 | 0.52 | 59 | 4993 | 7 (7) | 0 (0) | 0 (0) |
|  |  | Early decline | 4958 | 1.00 | 4958 | 3562 | 1121 (1121) | 248 (248) | 27 (27) |
|  |  | Late decline | 4993 | 0.52 | 30 | 4991 | 0 (0) | 2 (2) | 0 (0) |
|  |  | Intermediate decline | 4970 | 0.99 | 4698 | 3651 | 1118 (1118) | 177 (177) | 24 (24) |
|  |  | Intermediate decline (extra r.e.) | 5000 | 0.43 | 2 | 5000 | 0 (0) | 0 (0) | 0 (0) |
|  | non-proportional | Delayed decline | 5000 | 0.55 | 131 | 4982 | 12 (12) | 6 (6) | 0 (0) |
|  |  | Early decline | 4956 | 1.00 | 4956 | 3246 | 1363 (1363) | 317 (317) | 30 (30) |
|  |  | Late decline | 4993 | 0.51 | 23 | 4990 | 3 (3) | 0 (0) | 0 (0) |
|  |  | Intermediate decline | 4987 | 0.96 | 3977 | 4137 | 737 (737) | 100 (100) | 13 (13) |
|  |  | Intermediate decline (extra r.e.) | 5000 | 0.45 | 0 | 5000 | 0 (0) | 0 (0) | 0 (0) |
| NR = Newton-Raphson; DFP = Davidon-Fletcher-Powell; BFGS = Broyden-Fletcher-Goldfarb-Shanno; r.e. = random effect | | | | | | | | | |

| Table A4.3: Mean correlation between random intercepts and slopes for random slopes model; larger residual variance: observational studies | | | | | | | |
| --- | --- | --- | --- | --- | --- | --- | --- |
| Trajectory | Number converged | Mean correlation between intercepts and slopes | Number converged with correlation  > 0.99 or  < -0.99 | Number converged using NR algorithm | Number converged using DFP algorithm (and correlation  > 0.99 or  < -0.99) | Number converged using BFGS algorithm (and correlation  > 0.99 or  < -0.99) | Number converged using matlog option (and correlation  > 0.99 or  < -0.99) |
| Steady decline | 4994 | 0.56 | 1089 | 4904 | 80 (70) | 10 (9) | 0 (0) |
| Early decline | 4994 | 0.80 | 2745 | 4768 | 206 (191) | 20 (18) | 0 (0) |
| Late decline | 4996 | 0.80 | 2719 | 4775 | 205 (192) | 16 (11) | 0 (0) |
| Intermediate decline | 4999 | 0.64 | 1510 | 4858 | 124 (111) | 17 (15) | 0 (0) |
| NR = Newton-Raphson; DFP = Davidon-Fletcher-Powell; BFGS = Broyden-Fletcher-Goldfarb-Shanno; r.e. = random effect | | | | | | | |

| Table A4.4: Mean correlation between random intercepts and slopes for random slopes model; larger residual variance: trials | | | | | | | | | |
| --- | --- | --- | --- | --- | --- | --- | --- | --- | --- |
| Trial length (years) | Treatment | Trajectory | Number converged | Mean correlation between intercepts and slopes | Number converged with correlation  > 0.99 or  < -0.99 | Number converged using NR algorithm | Number converged using DFP algorithm (and correlation  > 0.99 or  < -0.99) | Number converged using BFGS algorithm (and correlation  > 0.99 or  < -0.99) | Number converged using matlog option (and correlation  > 0.99 or  < -0.99) |
| 5 | none | Steady decline | 4992 | 0.53 | 1795 | 4893 | 92 (89) | 7 (5) | 0 (0) |
|  |  | Early decline | 4993 | 0.70 | 2739 | 4835 | 152 (145) | 6 (6) | 0 (0) |
|  |  | Late decline | 4993 | 0.69 | 2713 | 4849 | 139 (136) | 5 (4) | 0 (0) |
|  |  | Intermediate decline | 4998 | 0.60 | 2170 | 4876 | 119 (111) | 3 (3) | 0 (0) |
|  | proportional | Steady decline | 4992 | 0.53 | 1854 | 4881 | 105 (102) | 6 (5) | 0 (0) |
|  |  | Early decline | 4993 | 0.69 | 2719 | 4834 | 150 (146) | 9 (8) | 0 (0) |
|  |  | Late decline | 4994 | 0.70 | 2772 | 4847 | 143 (141) | 4 (4) | 0 (0) |
|  |  | Intermediate decline | 4996 | 0.60 | 2121 | 4884 | 107 (103) | 5 (5) | 0 (0) |
|  | non-proportional | Delayed decline | 4993 | 0.53 | 1770 | 4874 | 113 (105) | 6 (6) | 0 (0) |
|  |  | Early decline | 4990 | 0.62 | 2326 | 4865 | 119 (117) | 6 (5) | 0 (0) |
|  |  | Late decline | 4995 | 0.63 | 2327 | 4880 | 105 (101) | 10 (8) | 0 (0) |
|  |  | Intermediate decline | 4999 | 0.58 | 2080 | 4857 | 132 (128) | 10 (9) | 0 (0) |
| 3 | none | Steady decline | 4989 | 0.50 | 2088 | 4911 | 71 (59) | 7 (6) | 0 (0) |
|  |  | Early decline | 4993 | 0.77 | 3614 | 4861 | 125 (116) | 7 (5) | 0 (0) |
|  |  | Late decline | 4995 | 0.51 | 2161 | 4885 | 99 (90) | 11 (3) | 0 (0) |
|  |  | Intermediate decline | 4994 | 0.65 | 2904 | 4866 | 118 (105) | 10 (6) | 0 (0) |
|  | proportional | Steady decline | 4986 | 0.50 | 2095 | 4883 | 89 (76) | 14 (12) | 0 (0) |
|  |  | Early decline | 4993 | 0.76 | 3548 | 4855 | 133 (116) | 5 (2) | 0 (0) |
|  |  | Late decline | 4992 | 0.51 | 2128 | 4898 | 88 (72) | 6 (4) | 0 (0) |
|  |  | Intermediate decline | 4996 | 0.65 | 2950 | 4881 | 108 (94) | 7 (5) | 0 (0) |
|  | non-proportional | Delayed decline | 4990 | 0.51 | 2124 | 4906 | 80 (71) | 4 (2) | 0 (0) |
|  |  | Early decline | 4988 | 0.67 | 3032 | 4864 | 110 (96) | 14 (10) | 0 (0) |
|  |  | Late decline | 4988 | 0.50 | 2122 | 4884 | 91 (81) | 13 (10) | 0 (0) |
|  |  | Intermediate decline | 4991 | 0.60 | 2628 | 4883 | 101 (86) | 7 (4) | 0 (0) |
| NR = Newton-Raphson; DFP = Davidon-Fletcher-Powell; BFGS = Broyden-Fletcher-Goldfarb-Shanno; r.e. = random effect | | | | | | | | | |

### A5. Results for intermediate decline trajectory with extra random effect


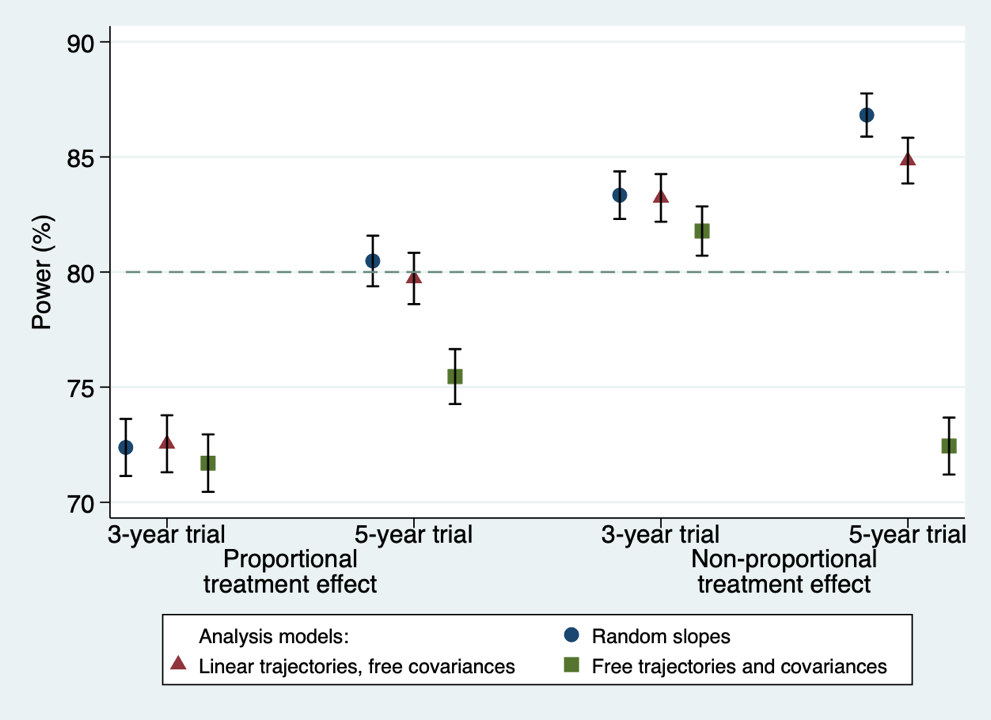


Figure A5.1: power for the intermediate decline trajectory with extra random effect, for 3- and 5-year trials. Left: treatment effects proportional to time. Right: treatment effects proportional to control group arm change. Ranges plotted are $\pm1.96$ times the Monte Carlo standard error. Results are summarised for datasets for which all models converged, within each scenario.

### A6. Results for scenarios with residual error variance $\boldsymbol{\sigma}_{\boldsymbol{e}}^{\boldsymbol{2}}\boldsymbol{=0.15}$

Within each trajectory type, results are summarised for datasets for which all models converged. Ranges plotted are $\pm1.96$ times the Monte Carlo standard error unless otherwise specified.

#### A6.1. No treatment effect

| Table A6.1.1: no treatment effect, 5-year trial with annual visits; RS = random slopes; LTFC = linear trajectories, free covariance; FTFC = free trajectories, free covariance | | | | | | | | | |
| --- | --- | --- | --- | --- | --- | --- | --- | --- | --- |
| Trajectory | Method of analysis | Number of simulations converged | Mean sample size for trial (SD; range) | Mean treatment effect^1^ | Empirical SE^1^ | Model-based SE^2^ | Percentage bias in model-based SE^3^ | Type I error |  |
| Steady decline | RS | 5000 | 230 (10; 190 to 270) | 0.000 | 0.089 | 0.089 | 0.4 | 5.2 |  |
|  | LTFC | 5000 |  | -0.000 | 0.090 | 0.093 | 3.2 | 4.5 |  |
|  | FTFC | 5000 |  | -0.000 | 0.098 | 0.099 | 1.1 | 4.8 |  |
| Early decline | RS | 4990 | 236 ( 8; 212 to 272) | 0.001 | 0.090 | 0.090 | 0.2 | 5.2 |  |
|  | LTFC | 4991 |  | 0.001 | 0.090 | 0.090 | -0.1 | 5.5 |  |
|  | FTFC | 4991 |  | 0.001 | 0.097 | 0.097 | -0.1 | 5.2 |  |
| Late decline | RS | 4991 | 236 ( 8; 212 to 270) | 0.000 | 0.088 | 0.090 | 1.9 | 4.6 |  |
|  | LTFC | 4993 |  | 0.000 | 0.089 | 0.088 | -0.4 | 5.1 |  |
|  | FTFC | 4993 |  | -0.001 | 0.097 | 0.097 | 0.1 | 4.7 |  |
| Intermediate decline | RS | 5000 | 230 (10; 198 to 264) | -0.000 | 0.090 | 0.089 | -0.4 | 5.0 |  |
|  | LTFC | 5000 |  | 0.000 | 0.091 | 0.093 | 2.4 | 4.4 |  |
|  | FTFC | 5000 |  | 0.000 | 0.097 | 0.099 | 1.6 | 5.0 |  |
| Intermediate decline (extra random effect) | RS | 5000 | 249 (11; 212 to 288) | -0.001 | 0.090 | 0.089 | -1.0 | 5.3 |  |
|  | LTFC | 5000 |  | -0.001 | 0.091 | 0.090 | -0.1 | 5.0 |  |
|  | FTFC | 5000 |  | -0.002 | 0.096 | 0.095 | -0.9 | 5.5 |  |
| 1. Treatment effect and SE at 5 years; empirical SE is SD of the treatment estimates across the simulations 2. Model-based SE is square root of the mean of the treatment effect variances across the simulations 3. (Model-based SE - Empirical SE)/Empirical SE x 100 | | | | | | | | | |


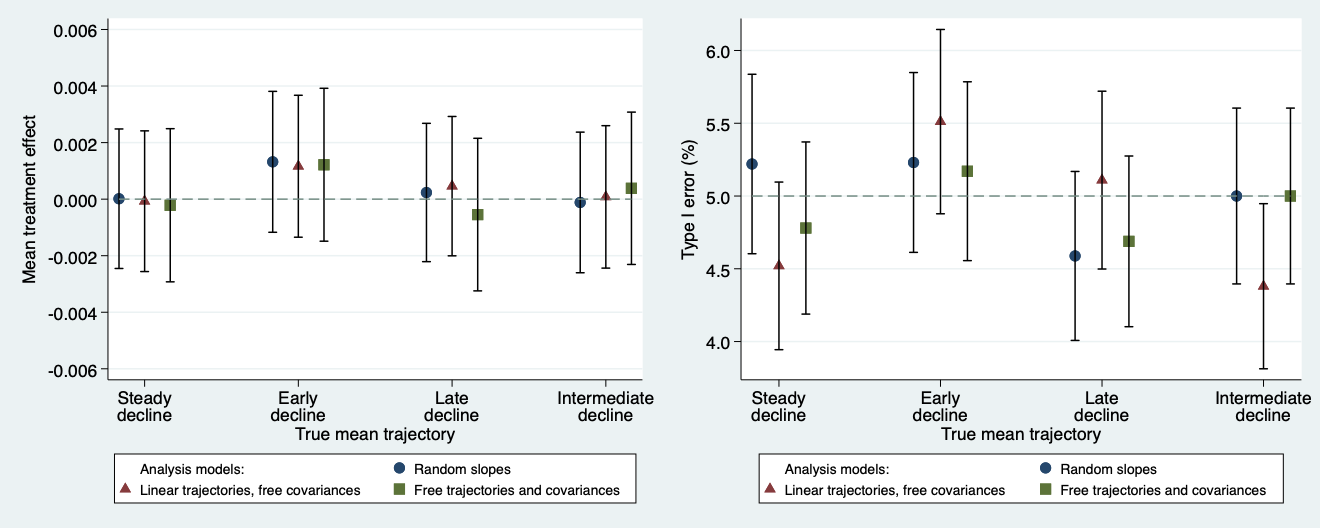


Figure A6.1.1: Mean treatment effect (left-hand panel) and Type I error (right-hand panel); 5-year trial, no treatment effect

| Table A6.1.2: no treatment effect, 5-year trial with annual visits, restricting to simulations in which the observational study has correlation of magnitude < 0.99; RS = random slopes; LTFC = linear trajectories, free covariance; FTFC = free trajectories, free covariance | | | | | | | | |
| --- | --- | --- | --- | --- | --- | --- | --- | --- |
| Trajectory | Method of analysis | Number of simulations converged | Mean sample size for trial (SD; range) | Mean treatment effect^1^ | Empirical SE^1^ | Model-based SE^2^ | Percentage bias in model-based SE^3^ | Type I error |
| Early decline | RS | 1195 | 240 ( 8; 214 to 272) | 0.001 | 0.088 | 0.089 | 1.6 | 4.9 |
|  | LTFC | 1195 |  | 0.001 | 0.088 | 0.090 | 1.4 | 4.9 |
|  | FTFC | 1195 |  | 0.001 | 0.097 | 0.097 | -0.6 | 5.1 |
| Late decline | RS | 1207 | 240 ( 8; 214 to 270) | -0.005 | 0.088 | 0.089 | 0.9 | 4.7 |
|  | LTFC | 1207 |  | -0.005 | 0.089 | 0.088 | -1.7 | 5.2 |
|  | FTFC | 1207 |  | -0.006 | 0.097 | 0.096 | -0.2 | 4.3 |
| 1. Treatment effect and SE at 5 years; empirical SE is SD of the treatment estimates across the simulations 2. Model-based SE is square root of the mean of the treatment effect variances across the simulations 3. (Model-based SE - Empirical SE)/Empirical SE x 100 | | | | | | | | |

| Table A6.1.3: no treatment effect, 3-year trial with annual visits; RS = random slopes; LTFC = linear trajectories, free covariance; FTFC = free trajectories, free covariance | | | | | | | | |
| --- | --- | --- | --- | --- | --- | --- | --- | --- |
| Trajectory | Method of analysis | Number of simulations converged | Mean sample size for trial (SD; range) | Mean treatment effect^1^ | Empirical SE^1^ | Model-based SE^2^ | Percentage bias in model-based SE^3^ | Type I error |
| Steady decline | RS | 5000 | 501 (12; 460 to 550) | 0.000 | 0.054 | 0.054 | -0.8 | 5.1 |
|  | LTFC | 5000 |  | 0.000 | 0.054 | 0.054 | -0.8 | 5.2 |
|  | FTFC | 5000 |  | -0.000 | 0.056 | 0.056 | -1.0 | 5.0 |
| Early decline | RS | 4969 | 661 (14; 610 to 714) | -0.001 | 0.046 | 0.051 | 12.0 | 2.7 |
|  | LTFC | 4991 |  | -0.001 | 0.046 | 0.047 | 1.8 | 4.9 |
|  | FTFC | 4991 |  | -0.001 | 0.047 | 0.048 | 2.2 | 4.5 |
| Late decline | RS | 4993 | 661 (14; 616 to 712) | -0.000 | 0.047 | 0.047 | -1.3 | 5.5 |
|  | LTFC | 4993 |  | -0.000 | 0.047 | 0.047 | -1.2 | 5.4 |
|  | FTFC | 4993 |  | -0.000 | 0.049 | 0.048 | -1.5 | 5.1 |
| Intermediate decline | RS | 4987 | 558 (13; 516 to 604) | -0.000 | 0.051 | 0.052 | 3.8 | 4.3 |
|  | LTFC | 5000 |  | -0.000 | 0.051 | 0.051 | 0.4 | 5.3 |
|  | FTFC | 5000 |  | -0.000 | 0.053 | 0.053 | 0.2 | 5.0 |
| Intermediate decline (extra random effect) | RS | 5000 | 585 (14; 536 to 632) | 0.002 | 0.057 | 0.058 | 1.2 | 5.0 |
|  | LTFC | 5000 |  | 0.002 | 0.057 | 0.058 | 1.2 | 4.9 |
|  | FTFC | 5000 |  | 0.002 | 0.058 | 0.058 | 1.2 | 5.0 |
| 1. Treatment effect and SE at 3 years; empirical SE is SD of the treatment estimates across the simulations 2. Model-based SE is square root of the mean of the treatment effect variances across the simulations 3. (Model-based SE - Empirical SE)/Empirical SE x 100 | | | | | | | | |


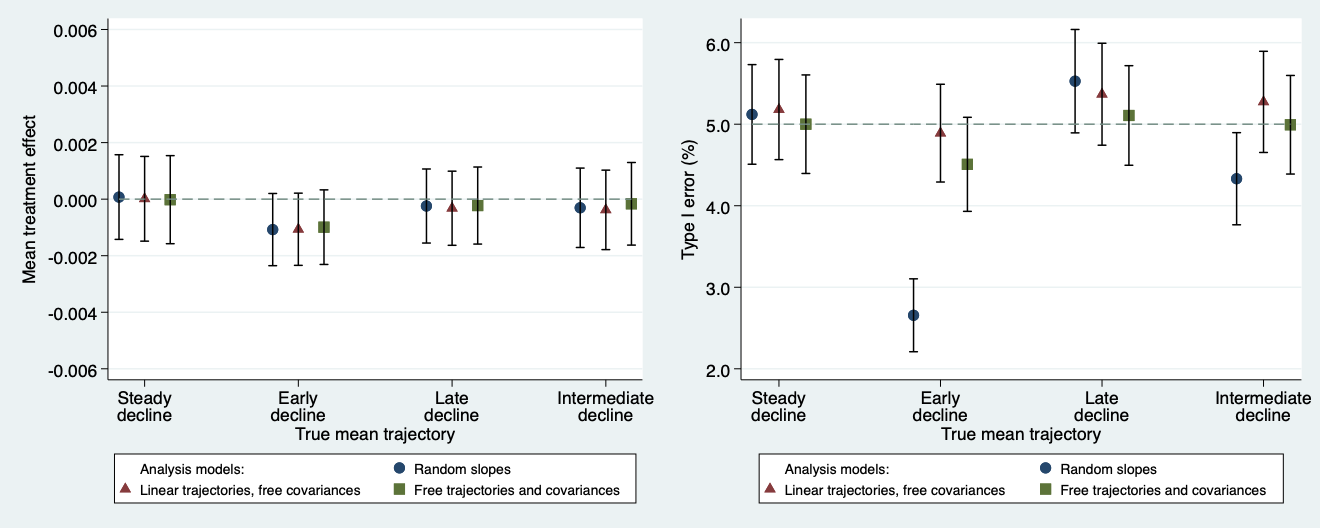


Figure A6.1.3: Mean treatment effect (left-hand panel) and Type I error (right-hand panel); 3-year trial, no treatment effect

| Table A6.1.4: no treatment effect, 3-year trial with annual visits, restricting to simulations in which the observational study has correlation of magnitude < 0.99; RS = random slopes; LTFC = linear trajectories, free covariance; FTFC = free trajectories, free covariance | | | | | | | | |
| --- | --- | --- | --- | --- | --- | --- | --- | --- |
| Trajectory | Method of analysis | Number of simulations converged | Mean sample size for trial (SD; range) | Mean treatment effect^1^ | Empirical SE^1^ | Model-based SE^2^ | Percentage bias in model-based SE^3^ | Type I error |
| Early decline | RS | 1188 | 662 (14; 610 to 714) | -0.002 | 0.045 | 0.051 | 13.0 | 3.0 |
|  | LTFC | 1195 |  | -0.002 | 0.045 | 0.047 | 2.7 | 5.0 |
|  | FTFC | 1195 |  | -0.001 | 0.047 | 0.048 | 2.6 | 4.5 |
| Late decline | RS | 1207 | 663 (14; 616 to 706) | 0.000 | 0.045 | 0.046 | 2.3 | 5.6 |
|  | LTFC | 1207 |  | 0.000 | 0.045 | 0.047 | 2.6 | 5.2 |
|  | FTFC | 1207 |  | -0.000 | 0.047 | 0.048 | 1.7 | 4.7 |
| 1. Treatment effect and SE at 3 years; empirical SE is SD of the treatment estimates across the simulations 2. Model-based SE is square root of the mean of the treatment effect variances across the simulations 3. (Model-based SE - Empirical SE)/Empirical SE x 100 | | | | | | | | |

#### A6.2 Treatment effect linearly proportional to time

| Table A6.2.1: linear treatment effect, 5-year trial with annual visits; RS = random slopes; LTFC = linear trajectories, free covariance; FCTFC = free control-group trajectories, free covariance; FTFC = free trajectories, free covariance | | | | | | | | |
| --- | --- | --- | --- | --- | --- | --- | --- | --- |
| Trajectory | Method of analysis | Number of simulations converged | Mean sample size for trial (SD; range) | Mean treatment effect^1^ | Empirical SE^1^ | Model-based SE^2^ | Percentage bias in model-based SE^3^ | Power |
| Steady decline | RS | 5000 | 230 (10; 190 to 270) | -0.252 | 0.089 | 0.089 | 0.3 | 80.1 |
|  | LTFC | 5000 |  | -0.252 | 0.090 | 0.093 | 2.9 | 77.9 |
|  | FCTFC | 5000 |  | -0.252 | 0.090 | 0.092 | 2.8 | 77.9 |
|  | FTFC | 5000 |  | -0.251 | 0.098 | 0.099 | 0.9 | 71.9 |
| Early decline | RS | 4987 | 236 ( 8; 212 to 272) | -0.249 | 0.088 | 0.090 | 1.7 | 78.4 |
|  | LTFC | 4991 |  | -0.249 | 0.089 | 0.090 | 1.3 | 78.0 |
|  | FTFC | 4991 |  | -0.249 | 0.098 | 0.097 | -0.8 | 71.2 |
| Late decline | RS | 4991 | 236 ( 8; 212 to 270) | -0.250 | 0.087 | 0.090 | 3.3 | 80.2 |
|  | LTFC | 4993 |  | -0.250 | 0.088 | 0.088 | 1.0 | 80.9 |
|  | FTFC | 4993 |  | -0.250 | 0.096 | 0.097 | 0.9 | 72.5 |
| Intermediate decline | RS | 5000 | 230 (10; 198 to 264) | -0.249 | 0.090 | 0.089 | -0.3 | 79.2 |
|  | LTFC | 5000 |  | -0.249 | 0.090 | 0.093 | 3.0 | 76.7 |
|  | FTFC | 5000 |  | -0.249 | 0.098 | 0.099 | 0.6 | 71.1 |
| Intermediate decline (extra random effect) | RS | 5000 | 249 (11; 212 to 288) | -0.252 | 0.089 | 0.089 | 0.3 | 80.5 |
|  | LTFC | 5000 |  | -0.253 | 0.090 | 0.090 | 1.1 | 79.7 |
|  | FTFC | 5000 |  | -0.252 | 0.096 | 0.095 | -0.9 | 75.5 |
| 1. Treatment effect and SE at 5 years; empirical SE is SD of the treatment estimates across the simulations 2. Model-based SE is square root of the mean of the treatment effect variances across the simulations 3. (Model-based SE - Empirical SE)/Empirical SE x 100 | | | | | | | | |


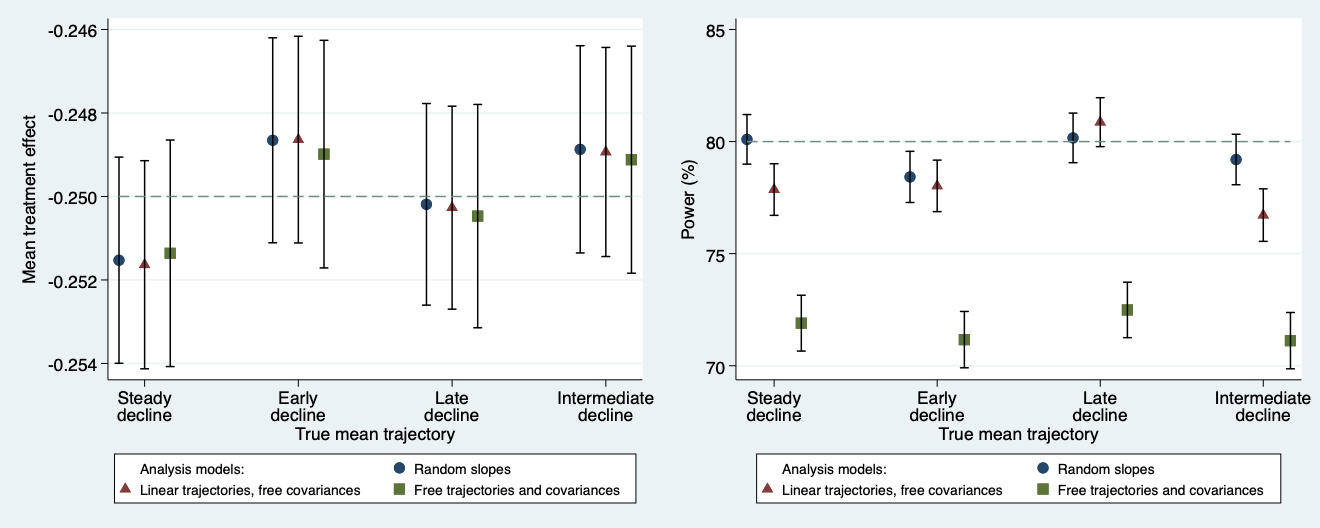


Figure A6.2.1: Mean treatment effect (left-hand panel) and Power (right-hand panel); 5-year trial, proportional treatment effect

| Table A6.2.2: linear treatment effect, 5-year trial with annual visits, restricting to simulations in which the observational study has correlation of magnitude < 0.99; RS = random slopes; LTFC = linear trajectories, free covariance; FTFC = free trajectories, free covariance | | | | | | | | |
| --- | --- | --- | --- | --- | --- | --- | --- | --- |
| Trajectory | Method of analysis | Number of simulations converged | Mean sample size for trial (SD; range) | Mean treatment effect^1^ | Empirical SE^1^ | Model-based SE^2^ | Percentage bias in model-based SE^3^ | Power |
| Early decline | RS | 1193 | 240 ( 8; 214 to 272) | -0.249 | 0.086 | 0.089 | 3.4 | 80.5 |
|  | LTFC | 1195 |  | -0.249 | 0.087 | 0.089 | 2.6 | 79.0 |
|  | FTFC | 1195 |  | -0.248 | 0.098 | 0.096 | -1.5 | 72.1 |
| Late decline | RS | 1205 | 240 ( 8; 214 to 270) | -0.248 | 0.088 | 0.089 | 1.4 | 80.5 |
|  | LTFC | 1207 |  | -0.247 | 0.089 | 0.088 | -1.2 | 80.9 |
|  | FTFC | 1207 |  | -0.248 | 0.097 | 0.096 | -1.0 | 72.5 |
| 1. Treatment effect and SE at 5 years; empirical SE is SD of the treatment estimates across the simulations 2. Model-based SE is square root of the mean of the treatment effect variances across the simulations 3. (Model-based SE - Empirical SE)/Empirical SE x 100 | | | | | | | | |

| Table A6.2.3: linear treatment effect, 3-year trial with annual visits; RS = random slopes; LTFC = linear trajectories, free covariance; FTFC = free trajectories, free covariance | | | | | | | | |
| --- | --- | --- | --- | --- | --- | --- | --- | --- |
| Trajectory | Method of analysis | Number of simulations converged | Mean sample size for trial (SD; range) | Mean treatment effect^1^ | Empirical SE^1^ | Model-based SE^2^ | Percentage bias in model-based SE^3^ | Power |
| Steady decline | RS | 5000 | 501 (12; 460 to 550) | -0.149 | 0.053 | 0.054 | 1.1 | 79.2 |
|  | LTFC | 5000 |  | -0.149 | 0.053 | 0.054 | 1.0 | 78.8 |
|  | FTFC | 5000 |  | -0.149 | 0.055 | 0.056 | 1.0 | 76.3 |
| Early decline | RS | 4958 | 661 (14; 610 to 714) | -0.149 | 0.046 | 0.051 | 10.7 | 84.6 |
|  | LTFC | 4991 |  | -0.150 | 0.046 | 0.047 | 0.5 | 89.4 |
|  | FTFC | 4991 |  | -0.150 | 0.048 | 0.048 | 0.8 | 86.8 |
| Late decline | RS | 4993 | 661 (14; 616 to 712) | -0.151 | 0.046 | 0.047 | 0.3 | 89.6 |
|  | LTFC | 4993 |  | -0.151 | 0.047 | 0.047 | 0.4 | 89.6 |
|  | FTFC | 4993 |  | -0.150 | 0.048 | 0.048 | 0.7 | 87.1 |
| Intermediate decline | RS | 4970 | 558 (13; 516 to 604) | -0.151 | 0.051 | 0.053 | 2.0 | 82.2 |
|  | LTFC | 5000 |  | -0.151 | 0.052 | 0.051 | -1.2 | 84.0 |
|  | FTFC | 5000 |  | -0.151 | 0.054 | 0.053 | -1.6 | 81.1 |
| Intermediate decline (extra random effect) | RS | 5000 | 585 (14; 536 to 632) | -0.148 | 0.057 | 0.058 | 0.4 | 72.4 |
|  | LTFC | 5000 |  | -0.148 | 0.057 | 0.058 | 0.4 | 72.5 |
|  | FTFC | 5000 |  | -0.148 | 0.058 | 0.058 | 0.6 | 71.7 |
| 1. Treatment effect and SE at 3 years; empirical SE is SD of the treatment estimates across the simulations 2. Model-based SE is square root of the mean of the treatment effect variances across the simulations 3. (Model-based SE - Empirical SE)/Empirical SE x 100 | | | | | | | | |


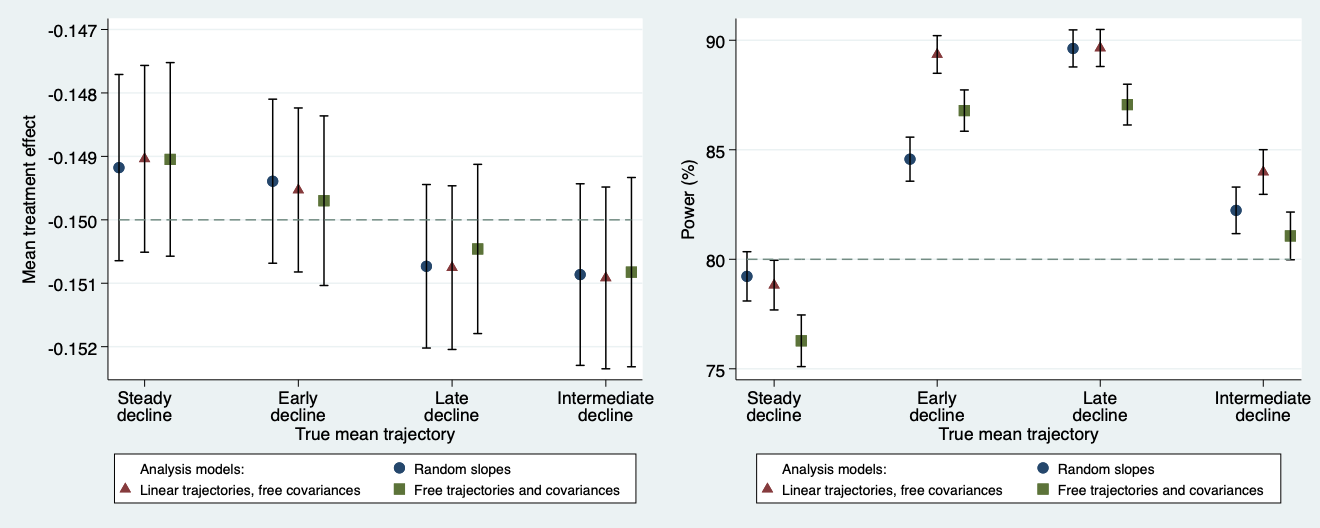


Figure A6.2.3: Mean treatment effect (left-hand panel) and Power (right-hand panel); 3-year trial, proportional treatment effect

| Table A6.2.4: linear treatment effect, 3-year trial with annual visits, restricting to simulations in which the observational study has correlation of magnitude < 0.99; RS = random slopes; LTFC = linear trajectories, free covariance; FTFC = free trajectories, free covariance | | | | | | | | |
| --- | --- | --- | --- | --- | --- | --- | --- | --- |
| Trajectory | Method of analysis | Number of simulations converged | Mean sample size for trial (SD; range) | Mean treatment effect^1^ | Empirical SE^1^ | Model-based SE^2^ | Percentage bias in model-based SE^3^ | Power |
| Early decline | RS | 1186 | 662 (14; 610 to 714) | -0.149 | 0.046 | 0.051 | 11.2 | 84.4 |
|  | LTFC | 1195 |  | -0.149 | 0.046 | 0.047 | 0.7 | 89.1 |
|  | FTFC | 1195 |  | -0.149 | 0.048 | 0.048 | 1.7 | 86.6 |
| Late decline | RS | 1207 | 663 (14; 616 to 706) | -0.152 | 0.046 | 0.047 | 0.9 | 90.9 |
|  | LTFC | 1207 |  | -0.152 | 0.046 | 0.047 | 0.9 | 90.6 |
|  | FTFC | 1207 |  | -0.152 | 0.047 | 0.048 | 2.1 | 88.3 |
| 1. Treatment effect and SE at 3 years; empirical SE is SD of the treatment estimates across the simulations 2. Model-based SE is square root of the mean of the treatment effect variances across the simulations 3. (Model-based SE - Empirical SE)/Empirical SE x 100 | | | | | | | | |

#### A6.3 Treatment effect not linearly proportional to time

| Table A6.3.1: non-linear treatment effect, 5-year trial with annual visits; RS = random slopes; LTFC = linear trajectories, free covariance; FTFC = free trajectories, free covariance | | | | | | | | |
| --- | --- | --- | --- | --- | --- | --- | --- | --- |
| Trajectory | Method of analysis | Number of simulations converged | Mean sample size for trial (SD; range) | Mean treatment effect^1^ | Empirical SE^1^ | Model-based SE^2^ | Percentage bias in model-based SE^3^ | Power |
| Delayed decline | RS | 5000 | 230 (10; 190 to 270) | -0.187 | 0.089 | 0.089 | 0.4 | 54.7 |
|  | LTFC | 5000 |  | -0.188 | 0.091 | 0.093 | 1.4 | 52.8 |
|  | FTFC | 5000 |  | -0.248 | 0.098 | 0.099 | 0.9 | 70.2 |
| Early decline | RS | 4991 | 236 ( 8; 212 to 272) | -0.181 | 0.087 | 0.089 | 2.2 | 53.3 |
|  | LTFC | 4991 |  | -0.181 | 0.090 | 0.091 | 1.0 | 51.8 |
|  | FTFC | 4991 |  | -0.250 | 0.095 | 0.097 | 2.1 | 73.3 |
| Late decline | RS | 4991 | 236 ( 8; 212 to 270) | -0.199 | 0.087 | 0.089 | 1.4 | 61.2 |
|  | LTFC | 4993 |  | -0.199 | 0.090 | 0.089 | -1.3 | 60.9 |
|  | FTFC | 4993 |  | -0.249 | 0.096 | 0.097 | 1.0 | 71.9 |
| Intermediate decline | RS | 5000 | 230 (10; 198 to 264) | -0.308 | 0.090 | 0.089 | -0.4 | 92.7 |
|  | LTFC | 5000 |  | -0.308 | 0.091 | 0.093 | 2.2 | 91.4 |
|  | FTFC | 5000 |  | -0.250 | 0.099 | 0.099 | -0.1 | 71.7 |
| Intermediate decline (extra random effect) | RS | 5000 | 249 (11; 212 to 288) | -0.273 | 0.088 | 0.089 | 0.4 | 86.8 |
|  | LTFC | 5000 |  | -0.267 | 0.089 | 0.090 | 0.9 | 84.8 |
|  | FTFC | 5000 |  | -0.242 | 0.095 | 0.095 | -0.8 | 72.4 |
| 1. Treatment effect and SE at 5 years; empirical SE is SD of the treatment estimates across the simulations 2. Model-based SE is square root of the mean of the treatment effect variances across the simulations 3. (Model-based SE - Empirical SE)/Empirical SE x 100 | | | | | | | | |


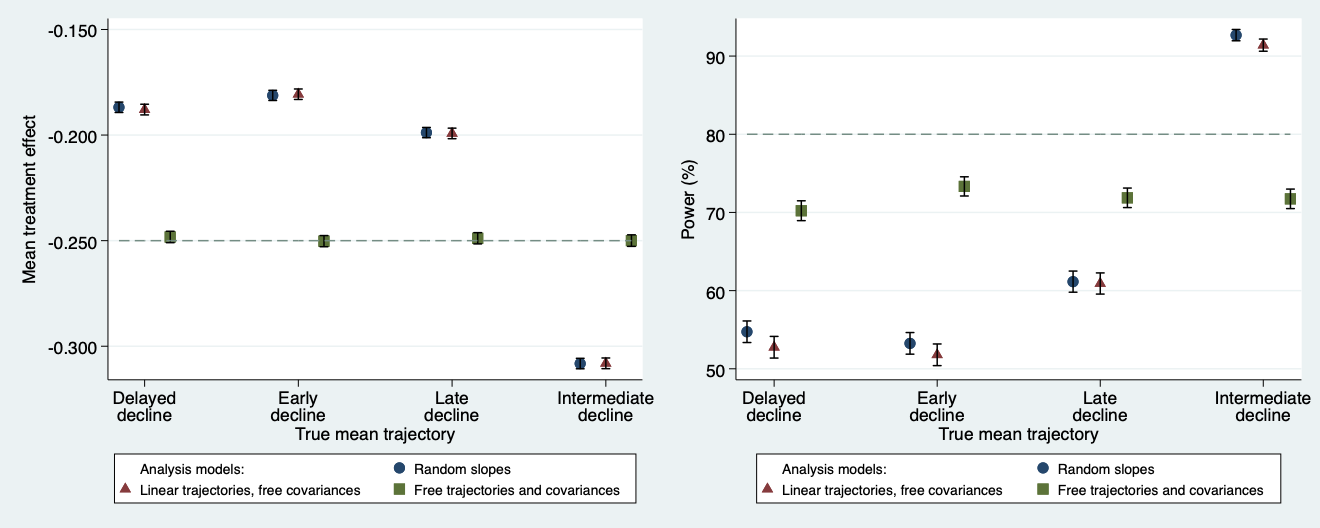


Figure A6.3.1: Mean treatment effect (left-hand panel) and Power (right-hand panel); 5-year trial, non-proportional treatment effect

| Table A6.3.2: non-linear treatment effect, 5-year trial, restricting to simulations in which observational study has correlation of magnitude < 0.99; RS = random slopes; LTFC = linear trajectories, free covariance; FTFC = free trajectories, free covariance | | | | | | | | |
| --- | --- | --- | --- | --- | --- | --- | --- | --- |
| Trajectory | Method of analysis | Number of simulations converged | Mean sample size for trial (SD; range) | Mean treatment effect^1^ | Empirical SE^1^ | Model-based SE^2^ | Percentage bias in model-based SE^3^ | Power |
| Early decline | RS | 1195 | 240 ( 8; 214 to 272) | -0.181 | 0.083 | 0.088 | 6.2 | 54.2 |
|  | LTFC | 1195 |  | -0.180 | 0.085 | 0.090 | 5.7 | 53.5 |
|  | FTFC | 1195 |  | -0.250 | 0.091 | 0.097 | 6.3 | 76.0 |
| Late decline | RS | 1207 | 240 ( 8; 214 to 270) | -0.198 | 0.086 | 0.088 | 2.0 | 60.9 |
|  | LTFC | 1207 |  | -0.199 | 0.090 | 0.088 | -1.6 | 60.9 |
|  | FTFC | 1207 |  | -0.249 | 0.096 | 0.097 | 0.4 | 71.6 |
| 1. Treatment effect and SE at 5 years; empirical SE is SD of the treatment estimates across the simulations 2. Model-based SE is square root of the mean of the treatment effect variances across the simulations 3. (Model-based SE - Empirical SE)/Empirical SE x 100 | | | | | | | | |

| Table A6.3.3: non-linear treatment effect, 3-year trial with annual visits; RS = random slopes; LTFC = linear trajectories, free covariance; FTFC = free trajectories, free covariance | | | | | | | | |
| --- | --- | --- | --- | --- | --- | --- | --- | --- |
| Trajectory | Method of analysis | Number of simulations converged | Mean sample size for trial (SD; range) | Mean treatment effect^1^ | Empirical SE^1^ | Model-based SE^2^ | Percentage bias in model-based SE^3^ | Power |
| Delayed decline | RS | 5000 | 501 (12; 460 to 550) | -0.242 | 0.055 | 0.054 | -1.9 | 99.4 |
|  | LTFC | 5000 |  | -0.242 | 0.055 | 0.054 | -2.7 | 99.3 |
|  | FTFC | 5000 |  | -0.249 | 0.056 | 0.056 | -1.1 | 99.4 |
| Early decline | RS | 4956 | 661 (14; 610 to 714) | -0.239 | 0.047 | 0.050 | 6.3 | 99.9 |
|  | LTFC | 4991 |  | -0.236 | 0.048 | 0.047 | -1.8 | 99.9 |
|  | FTFC | 4991 |  | -0.250 | 0.049 | 0.048 | -0.2 | 99.9 |
| Late decline | RS | 4993 | 661 (14; 616 to 712) | -0.004 | 0.047 | 0.047 | -1.4 | 5.3 |
|  | LTFC | 4993 |  | -0.004 | 0.047 | 0.047 | -1.3 | 5.2 |
|  | FTFC | 4993 |  | -0.004 | 0.049 | 0.048 | -1.0 | 5.4 |
| Intermediate decline | RS | 4987 | 558 (13; 516 to 604) | -0.195 | 0.051 | 0.052 | 1.4 | 96.4 |
|  | LTFC | 5000 |  | -0.195 | 0.051 | 0.051 | -0.8 | 96.6 |
|  | FTFC | 5000 |  | -0.204 | 0.053 | 0.053 | -0.2 | 97.2 |
| Intermediate decline (extra random effect) | RS | 5000 | 585 (14; 536 to 632) | -0.165 | 0.056 | 0.056 | 0.2 | 83.3 |
|  | LTFC | 5000 |  | -0.164 | 0.056 | 0.056 | 0.1 | 83.2 |
|  | FTFC | 5000 |  | -0.164 | 0.057 | 0.057 | 0.7 | 81.8 |
| 1. Treatment effect and SE at 3 years; empirical SE is SD of the treatment estimates across the simulations 2. Model-based SE is square root of the mean of the treatment effect variances across the simulations 3. (Model-based SE - Empirical SE)/Empirical SE x 100 | | | | | | | | |


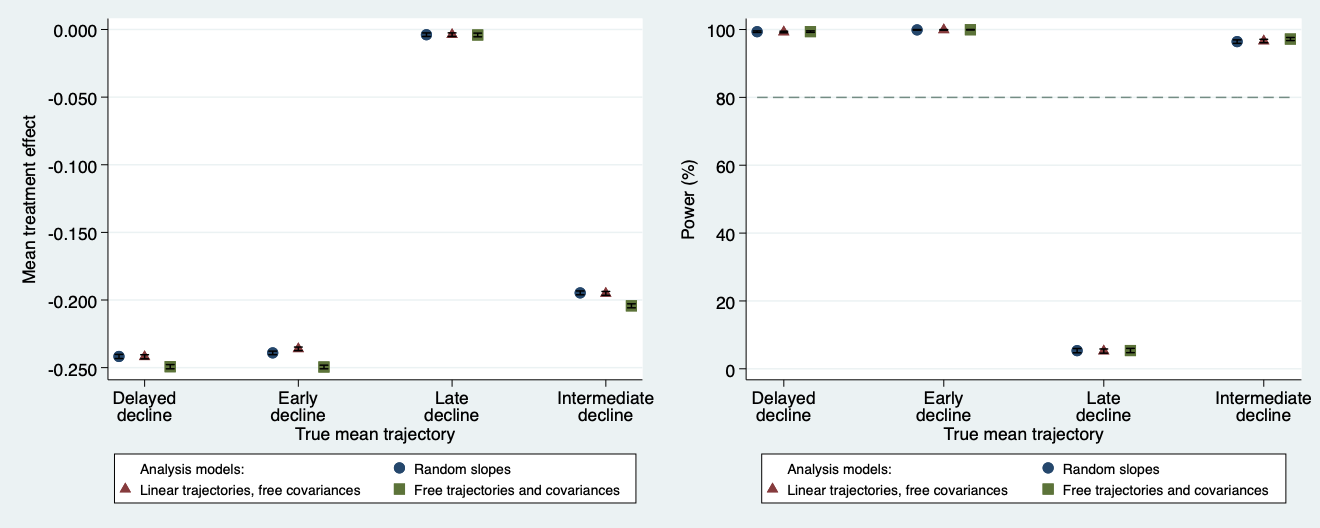


Figure A6.3.3: Mean treatment effect (left-hand panel) and Power (right-hand panel); 3-year trial, non-proportional treatment effect

| Table A6.3.4: non-linear treatment effect, 3-year trial with annual visits, restricting to simulations in which the observational study has correlation of magnitude < 0.99; RS = random slopes; LTFC = linear trajectories, free covariance; FTFC = free trajectories, free covariance | | | | | | | | |
| --- | --- | --- | --- | --- | --- | --- | --- | --- |
| Trajectory | Method of analysis | Number of simulations converged | Mean sample size for trial (SD; range) | Mean treatment effect^1^ | Empirical SE^1^ | Model-based SE^2^ | Percentage bias in model-based SE^3^ | Power |
| Early decline | RS | 1188 | 662 (15; 610 to 714) | -0.239 | 0.046 | 0.050 | 8.7 | 100.0 |
|  | LTFC | 1195 |  | -0.236 | 0.047 | 0.047 | 0.2 | 99.9 |
|  | FTFC | 1195 |  | -0.249 | 0.048 | 0.048 | 1.5 | 99.9 |
| Late decline | RS | 1207 | 663 (14; 616 to 706) | -0.002 | 0.046 | 0.047 | 1.5 | 5.0 |
|  | LTFC | 1207 |  | -0.002 | 0.046 | 0.047 | 1.6 | 4.6 |
|  | FTFC | 1207 |  | -0.003 | 0.048 | 0.048 | 1.7 | 5.5 |
| 1. Treatment effect and SE at 3 years; empirical SE is SD of the treatment estimates across the simulations 2. Model-based SE is square root of the mean of the treatment effect variances across the simulations 3. (Model-based SE - Empirical SE)/Empirical SE x 100 | | | | | | | | |

### A7. Results for scenarios with $\boldsymbol{\sigma}_{\boldsymbol{e}}^{\boldsymbol{2}}\boldsymbol{=2}$

#### A7.1 No treatment effect

| Table A7.1.1: greater residual variance, no treatment effect, 5-year trial with annual visits; RS = random slopes; LTFC = linear trajectories, free covariance; FTFC = free trajectories, free covariance | | | | | | | | |
| --- | --- | --- | --- | --- | --- | --- | --- | --- |
| Trajectory | Method of analysis | Number of simulations converged | Mean sample size for trial (SD; range) | Mean treatment effect^1^ | Empirical SE^1^ | Model-based SE^2^ | Percentage bias in model-based SE^3^ | Type I error |
| Steady decline | RS | 4992 | 264 (11; 226 to 310) | -0.001 | 0.179 | 0.179 | -0.1 | 4.7 |
|  | LTFC | 4994 |  | -0.001 | 0.181 | 0.180 | -0.4 | 4.9 |
|  | FTFC | 4994 |  | -0.003 | 0.210 | 0.211 | 0.3 | 4.9 |
| Early decline | RS | 4993 | 266 (11; 228 to 310) | -0.002 | 0.174 | 0.179 | 3.3 | 4.6 |
|  | LTFC | 4994 |  | -0.002 | 0.175 | 0.179 | 2.3 | 4.6 |
|  | FTFC | 4994 |  | -0.002 | 0.208 | 0.210 | 1.0 | 4.9 |
| Late decline | RS | 4993 | 265 (11; 230 to 316) | -0.002 | 0.178 | 0.180 | 1.0 | 4.7 |
|  | LTFC | 4996 |  | -0.002 | 0.179 | 0.180 | 0.3 | 4.8 |
|  | FTFC | 4996 |  | -0.002 | 0.211 | 0.210 | -0.5 | 4.9 |
| Intermediate decline | RS | 4998 | 265 (11; 230 to 314) | 0.002 | 0.176 | 0.179 | 1.7 | 4.8 |
|  | LTFC | 4999 |  | 0.002 | 0.178 | 0.180 | 0.9 | 5.0 |
|  | FTFC | 4999 |  | -0.002 | 0.210 | 0.211 | 0.2 | 5.3 |
| 1. Treatment effect and SE at 5 years; empirical SE is SD of the treatment estimates across the simulations 2. Model-based SE is square root of the mean of the treatment effect variances across the simulations 3. (Model-based SE - Empirical SE)/Empirical SE x 100 | | | | | | | | |


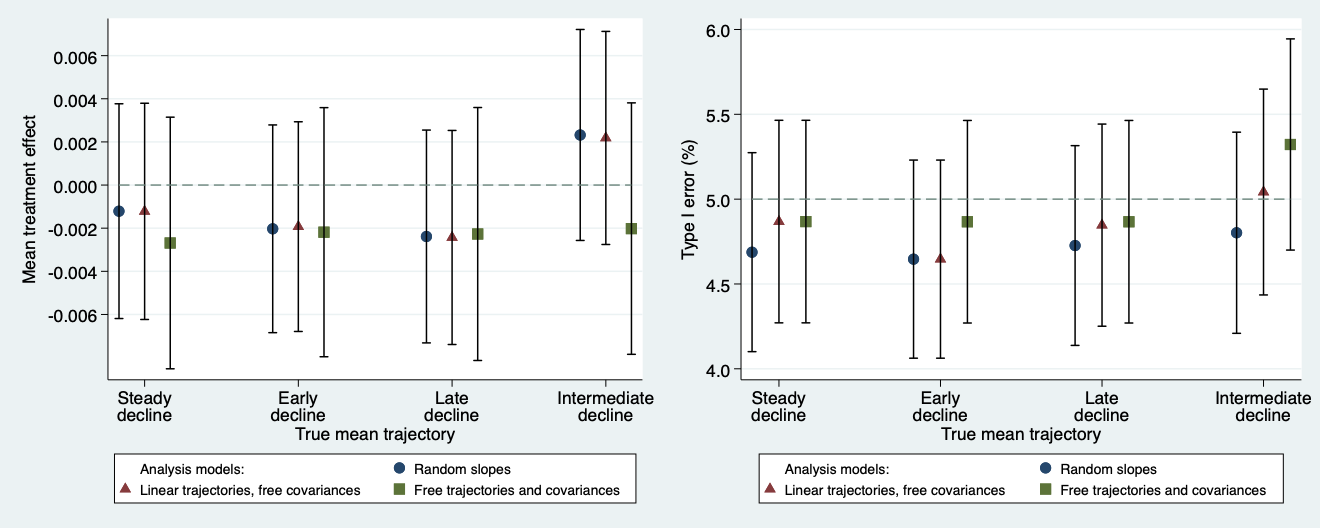


Figure A7.1.1: Mean treatment effect (left-hand panel) and Type I error (right-hand panel); 5-year trial, no treatment effect, larger residual error variance

| Table A7.1.2: greater residual variance, no treatment effect, 3-year trial with annual visits; RS = random slopes; LTFC = linear trajectories, free covariance; FTFC = free trajectories, free covariance | | | | | | | | |
| --- | --- | --- | --- | --- | --- | --- | --- | --- |
| Trajectory | Method of analysis | Number of simulations converged | Mean sample size for trial (SD; range) | Mean treatment effect^1^ | Empirical SE^1^ | Model-based SE^2^ | Percentage bias in model-based SE^3^ | Type I error |
| Steady decline | RS | 4989 | 761 (18; 698 to 838) | -0.000 | 0.107 | 0.107 | 0.0 | 4.9 |
|  | LTFC | 4994 |  | -0.000 | 0.108 | 0.107 | -0.2 | 5.1 |
|  | FTFC | 4994 |  | -0.000 | 0.118 | 0.118 | -0.1 | 4.8 |
| Early decline | RS | 4993 | 778 (18; 712 to 848) | -0.000 | 0.106 | 0.107 | 1.0 | 4.8 |
|  | LTFC | 4994 |  | -0.000 | 0.106 | 0.106 | 0.3 | 5.0 |
|  | FTFC | 4994 |  | 0.000 | 0.116 | 0.117 | 1.0 | 4.8 |
| Late decline | RS | 4995 | 778 (18; 712 to 864) | -0.002 | 0.107 | 0.106 | -0.9 | 5.2 |
|  | LTFC | 4996 |  | -0.002 | 0.107 | 0.106 | -1.1 | 5.2 |
|  | FTFC | 4996 |  | -0.002 | 0.118 | 0.117 | -1.1 | 5.4 |
| Intermediate decline | RS | 4994 | 767 (18; 700 to 824) | 0.001 | 0.106 | 0.107 | 1.2 | 4.8 |
|  | LTFC | 4999 |  | 0.001 | 0.106 | 0.107 | 0.6 | 4.9 |
|  | FTFC | 4999 |  | 0.001 | 0.116 | 0.118 | 1.5 | 4.7 |
| 1. Treatment effect and SE at 3 years; empirical SE is SD of the treatment estimates across the simulations 2. Model-based SE is square root of the mean of the treatment effect variances across the simulations 3. (Model-based SE - Empirical SE)/Empirical SE x 100 | | | | | | | | |


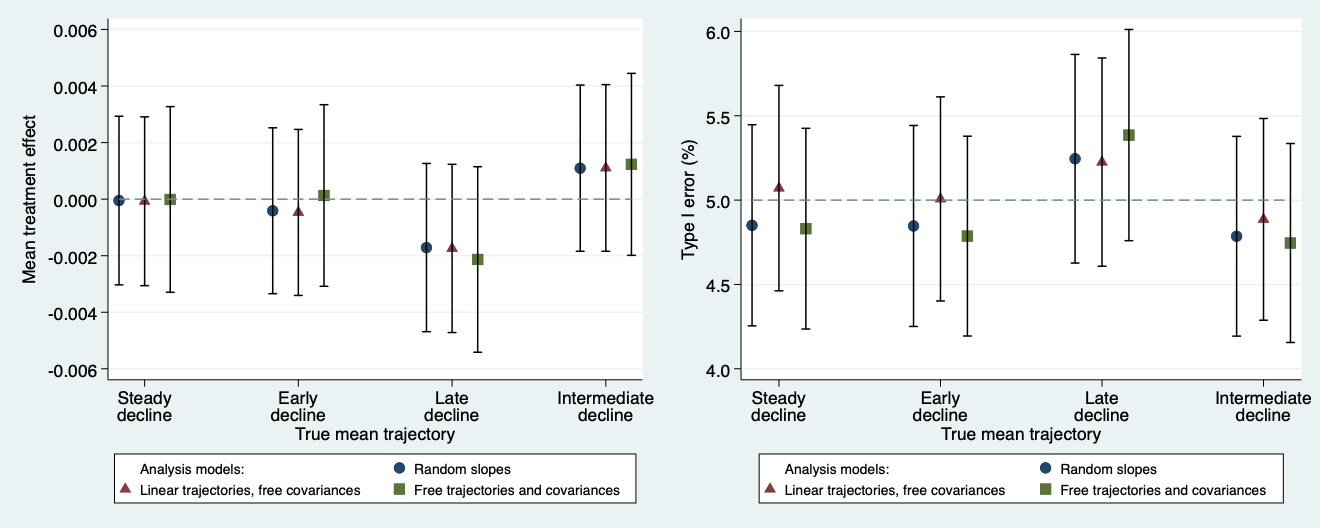


Figure A7.1.2: Mean treatment effect (left-hand panel) and Type I error (right-hand panel); 3-year trial, no treatment effect, larger residual error variance

#### A7.2 Proportional treatment effect

| Table A7.2.1: greater residual variance, proportional treatment effect, 5-year trial with annual visits; RS = random slopes; LTFC = linear trajectories, free covariance; FTFC = free trajectories, free covariance | | | | | | | | |
| --- | --- | --- | --- | --- | --- | --- | --- | --- |
| Trajectory | Method of analysis | Number of simulations converged | Mean sample size for trial (SD; range) | Mean treatment effect^1^ | Empirical SE^1^ | Model-based SE^2^ | Percentage bias in model-based SE^3^ | Power |
| Steady decline | RS | 4992 | 264 (11; 226 to 310) | -0.499 | 0.179 | 0.179 | 0.3 | 79.1 |
|  | LTFC | 4994 |  | -0.499 | 0.180 | 0.180 | -0.2 | 79.4 |
|  | FTFC | 4994 |  | -0.499 | 0.212 | 0.211 | -0.3 | 65.6 |
| Early decline | RS | 4993 | 266 (11; 228 to 310) | -0.497 | 0.179 | 0.180 | 0.4 | 78.9 |
|  | LTFC | 4994 |  | -0.496 | 0.180 | 0.179 | -0.3 | 78.8 |
|  | FTFC | 4994 |  | -0.495 | 0.209 | 0.210 | 0.4 | 65.0 |
| Late decline | RS | 4994 | 265 (11; 230 to 316) | -0.500 | 0.179 | 0.180 | 0.3 | 80.1 |
|  | LTFC | 4996 |  | -0.500 | 0.181 | 0.179 | -0.8 | 79.9 |
|  | FTFC | 4996 |  | -0.496 | 0.212 | 0.210 | -1.0 | 65.4 |
| Intermediate decline | RS | 4996 | 265 (11; 230 to 314) | -0.503 | 0.179 | 0.180 | 0.2 | 79.5 |
|  | LTFC | 4999 |  | -0.503 | 0.180 | 0.180 | -0.2 | 79.2 |
|  | FTFC | 4999 |  | -0.505 | 0.212 | 0.211 | -0.4 | 66.6 |
| 1. Treatment effect and SE at 5 years; empirical SE is SD of the treatment estimates across the simulations 2. Model-based SE is square root of the mean of the treatment effect variances across the simulations 3. (Model-based SE - Empirical SE)/Empirical SE x 100 | | | | | | | | |


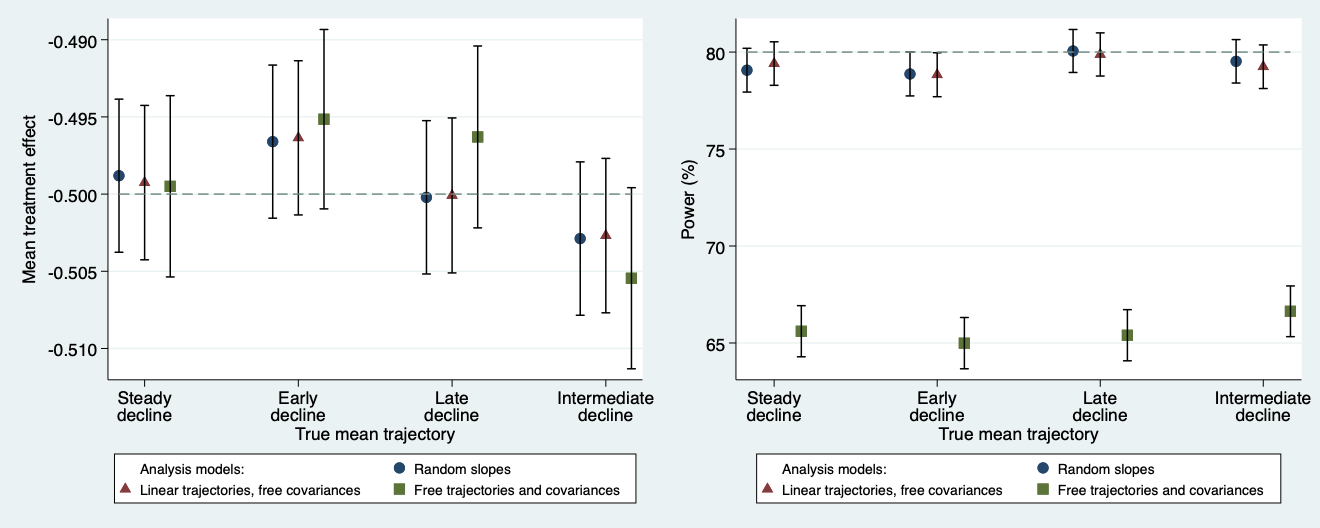


Figure A7.2.1: Mean treatment effect (left) and Power (right); 5-year trial, proportional treatment effect, larger residual variance

| Table A7.2.2: greater residual variance, proportional treatment effect, 3-year trial with annual visits; RS = random slopes; LTFC = linear trajectories, free covariance; FTFC = free trajectories, free covariance | | | | | | | | |
| --- | --- | --- | --- | --- | --- | --- | --- | --- |
| Trajectory | Method of analysis | Number of simulations converged | Mean sample size for trial (SD; range) | Mean treatment effect^1^ | Empirical SE^1^ | Model-based SE^2^ | Percentage bias in model-based SE^3^ | Power |
| Steady decline | RS | 4986 | 761 (18; 698 to 838) | -0.300 | 0.107 | 0.108 | 0.7 | 79.9 |
|  | LTFC | 4994 |  | -0.300 | 0.107 | 0.107 | 0.4 | 80.0 |
|  | FTFC | 4994 |  | -0.300 | 0.118 | 0.118 | 0.4 | 71.7 |
| Early decline | RS | 4993 | 778 (18; 712 to 848) | -0.299 | 0.107 | 0.107 | -0.5 | 79.9 |
|  | LTFC | 4994 |  | -0.299 | 0.108 | 0.106 | -1.3 | 80.2 |
|  | FTFC | 4994 |  | -0.298 | 0.118 | 0.117 | -1.0 | 72.5 |
| Late decline | RS | 4992 | 778 (18; 712 to 864) | -0.299 | 0.105 | 0.106 | 1.6 | 80.9 |
|  | LTFC | 4996 |  | -0.300 | 0.105 | 0.106 | 1.2 | 80.8 |
|  | FTFC | 4996 |  | -0.299 | 0.116 | 0.117 | 1.2 | 72.7 |
| Intermediate decline | RS | 4996 | 767 (18; 700 to 824) | -0.300 | 0.104 | 0.107 | 3.3 | 80.0 |
|  | LTFC | 4999 |  | -0.300 | 0.104 | 0.107 | 2.8 | 80.2 |
|  | FTFC | 4999 |  | -0.301 | 0.115 | 0.118 | 2.4 | 72.8 |
| 1. Treatment effect and SE at 3 years; empirical SE is SD of the treatment estimates across the simulations 2. Model-based SE is square root of the mean of the treatment effect variances across the simulations 3. (Model-based SE - Empirical SE)/Empirical SE x 100 | | | | | | | | |


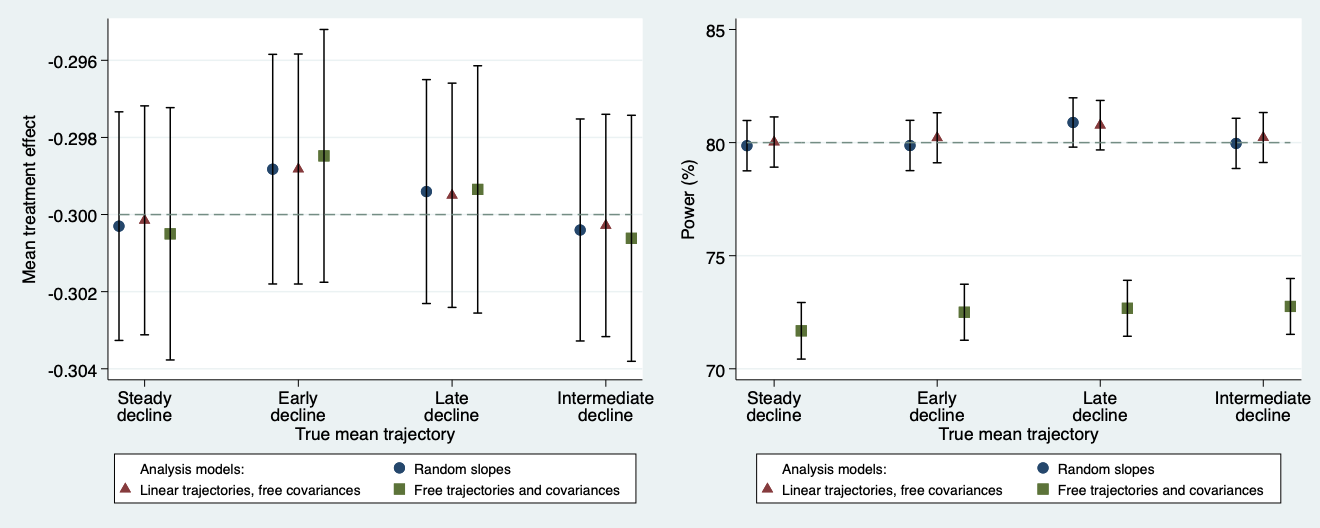


Figure A7.2.2: Mean treatment effect (left) and Power (right); 3-year trial, proportional treatment effect, larger residual variance

#### A7.3 Treatment effect not proportional to time

| Table A7.3.1: greater residual variance, non-proportional treatment effect, 5-year trial with annual visits; RS = random slopes; LTFC = linear trajectories, free covariance; FTFC = free trajectories, free covariance | | | | | | | | |
| --- | --- | --- | --- | --- | --- | --- | --- | --- |
| Trajectory | Method of analysis | Number of simulations converged | Mean sample size for trial (SD; range) | Mean treatment effect^1^ | Empirical SE^1^ | Model-based SE^2^ | Percentage bias in model-based SE^3^ | Power |
| Delayed decline | RS | 4993 | 264 (11; 226 to 310) | -0.580 | 0.179 | 0.179 | 0.3 | 90.0 |
|  | LTFC | 4994 |  | -0.579 | 0.181 | 0.180 | -0.3 | 89.8 |
|  | FTFC | 4994 |  | -0.499 | 0.209 | 0.211 | 1.0 | 65.7 |
| Early decline | RS | 4990 | 266 (11; 228 to 310) | -0.562 | 0.175 | 0.179 | 2.1 | 88.4 |
|  | LTFC | 4994 |  | -0.558 | 0.178 | 0.179 | 1.0 | 87.9 |
|  | FTFC | 4994 |  | -0.500 | 0.207 | 0.210 | 1.5 | 65.9 |
| Late decline | RS | 4960 | 265 (11; 230 to 316) | -0.306 | 0.176 | 0.179 | 1.7 | 39.4 |
|  | LTFC | 4961 |  | -0.308 | 0.179 | 0.179 | 0.3 | 39.7 |
|  | FTFC | 4963 |  | -0.500 | 0.207 | 0.210 | 1.4 | 66.5 |
| Intermediate decline | RS | 4999 | 265 (11; 230 to 314) | -0.569 | 0.178 | 0.179 | 0.6 | 88.5 |
|  | LTFC | 4999 |  | -0.570 | 0.180 | 0.180 | -0.2 | 88.5 |
|  | FTFC | 4999 |  | -0.504 | 0.212 | 0.211 | -0.6 | 66.0 |
| 1. Treatment effect and SE at 5 years; empirical SE is SD of the treatment estimates across the simulations 2. Model-based SE is square root of the mean of the treatment effect variances across the simulations 3. (Model-based SE - Empirical SE)/Empirical SE x 100 | | | | | | | | |


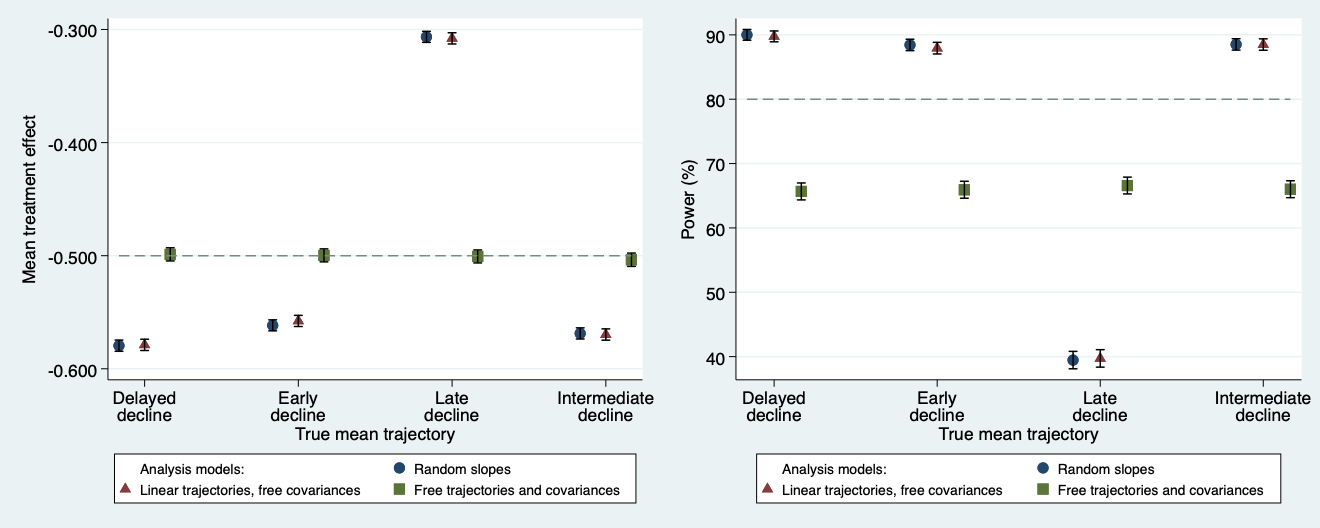


Figure A7.3.1: Mean treatment effect (left-hand panel) and Power (right-hand panel); 5-year trial, non-proportional treatment effect, larger residual error variance

| Table A7.3.2: greater residual variance, non-proportional treatment effect, 3-year trial with annual visits; RS = random slopes; LTFC = linear trajectories, free covariance; FTFC = free trajectories, free covariance | | | | | | | | |
| --- | --- | --- | --- | --- | --- | --- | --- | --- |
| Trajectory | Method of analysis | Number of simulations converged | Mean sample size for trial (SD; range) | Mean treatment effect^1^ | Empirical SE^1^ | Model-based SE^2^ | Percentage bias in model-based SE^3^ | Power |
| Delayed decline | RS | 4990 | 761 (18; 698 to 838) | -0.529 | 0.106 | 0.108 | 1.6 | 99.9 |
|  | LTFC | 4994 |  | -0.528 | 0.106 | 0.107 | 1.3 | 99.9 |
|  | FTFC | 4994 |  | -0.501 | 0.118 | 0.118 | 0.1 | 98.8 |
| Early decline | RS | 4988 | 778 (18; 712 to 848) | -0.578 | 0.107 | 0.107 | -0.5 | 100.0 |
|  | LTFC | 4994 |  | -0.576 | 0.107 | 0.106 | -1.3 | 100.0 |
|  | FTFC | 4994 |  | -0.500 | 0.118 | 0.117 | -1.1 | 98.9 |
| Late decline | RS | 4988 | 778 (18; 712 to 864) | -0.008 | 0.106 | 0.106 | 0.5 | 5.1 |
|  | LTFC | 4996 |  | -0.008 | 0.106 | 0.106 | 0.1 | 5.2 |
|  | FTFC | 4996 |  | -0.011 | 0.116 | 0.117 | 0.5 | 5.1 |
| Intermediate decline | RS | 4991 | 767 (18; 700 to 824) | -0.330 | 0.104 | 0.107 | 3.1 | 87.1 |
|  | LTFC | 4999 |  | -0.330 | 0.105 | 0.107 | 2.1 | 87.2 |
|  | FTFC | 4999 |  | -0.409 | 0.115 | 0.118 | 2.1 | 93.9 |
| 1. Treatment effect and SE at 3 years; empirical SE is SD of the treatment estimates across the simulations 2. Model-based SE is square root of the mean of the treatment effect variances across the simulations 3. (Model-based SE - Empirical SE)/Empirical SE x 100 | | | | | | | | |


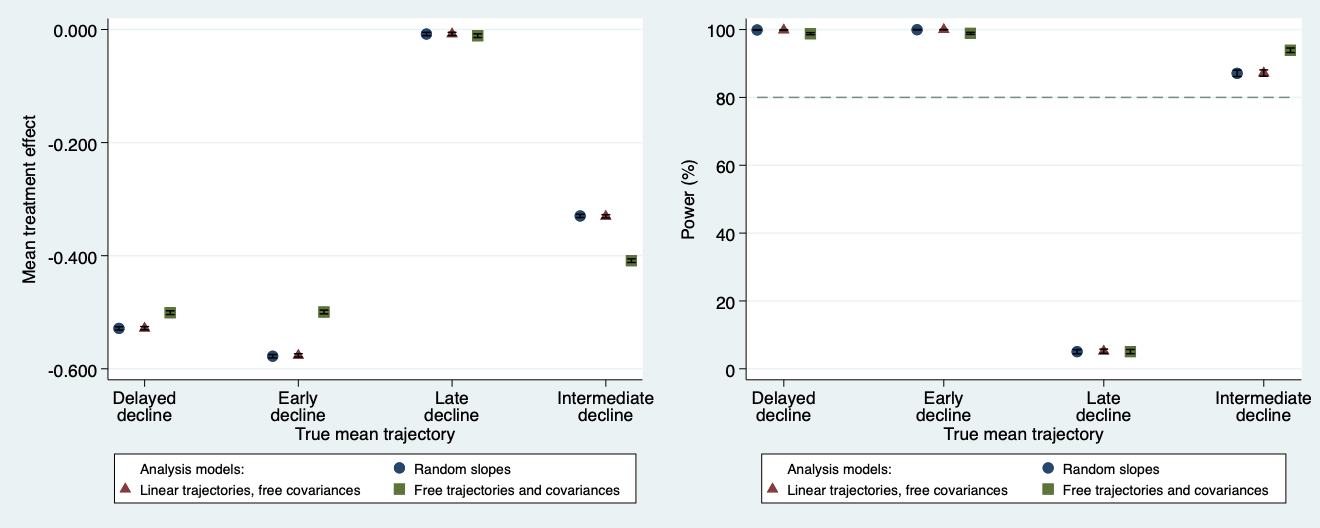


Figure A7.3.2: Mean treatment effect (left-hand panel) and Power (right-hand panel); 3-year trial, non-proportional treatment effect, larger residual error variance

### A8. Values used in sample size calculation estimated from the observational studies

#### A8.1 Residual error $\boldsymbol{\sigma}_{\boldsymbol{e}}^{\boldsymbol{2}}\boldsymbol{=0.15}$


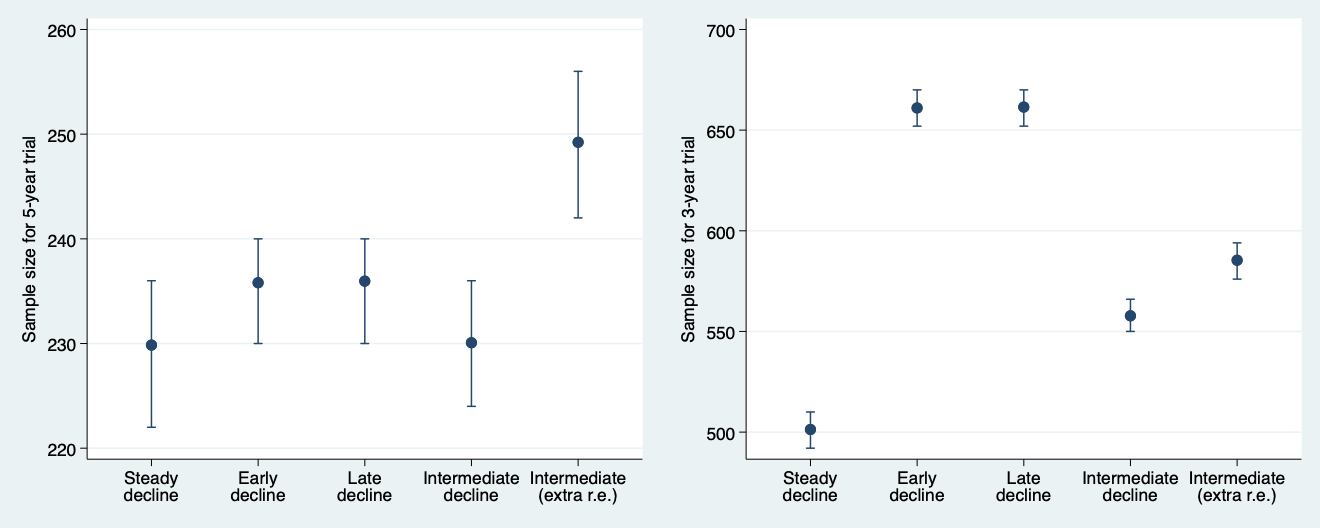


Figure A8.1.1: Mean sample sizes and IQR predicted by observational studies for 5-year trial (left) and 3-year trial (right)


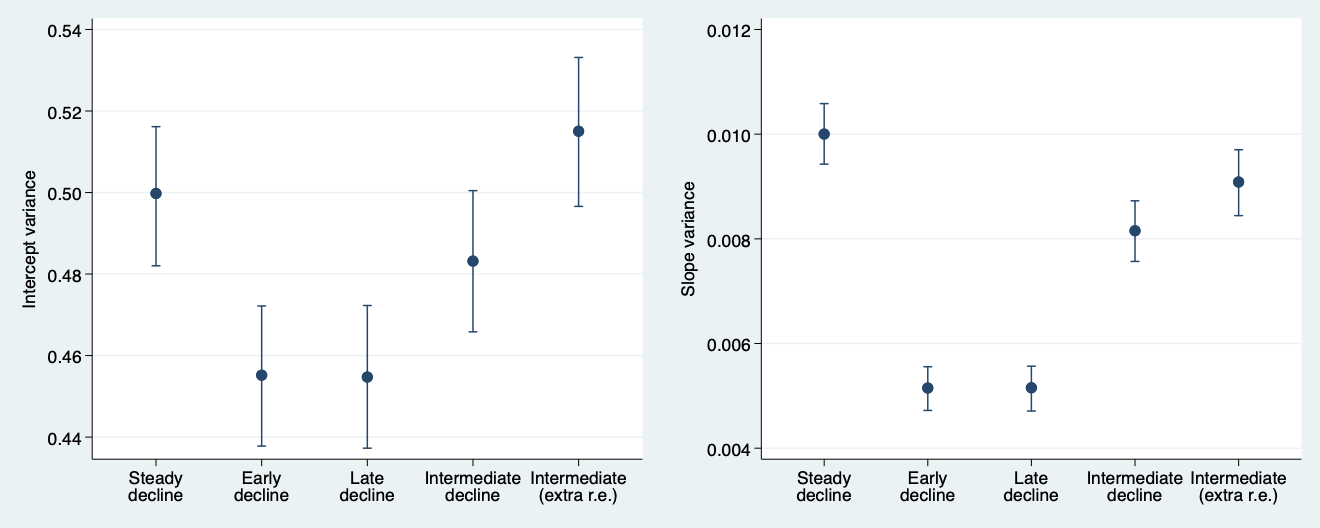


Figure A8.1.2: Mean intercept (left) and slope (right-hand panel) variance and IQR predicted by observational studies


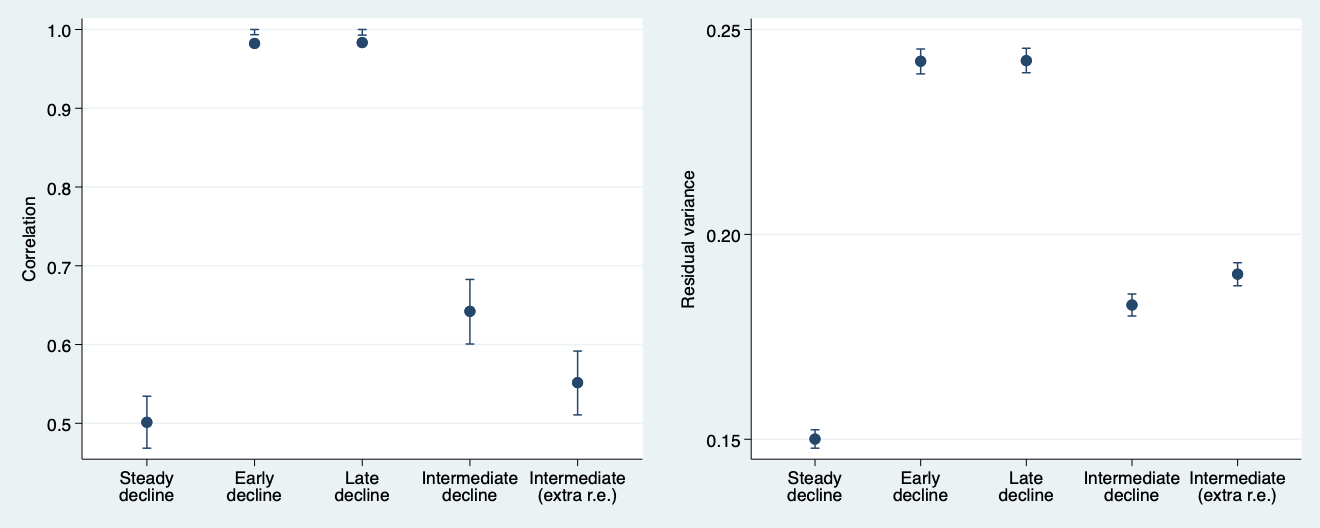


Figure A8.1.3: Mean correlation between random intercepts and slopes (left-hand panel) and mean residual variance (right-hand panel) and IQR predicted by observational studies

#### A8.2 Residual variance $\boldsymbol{\sigma}_{\boldsymbol{e}}^{\boldsymbol{2}}\boldsymbol{=2}$


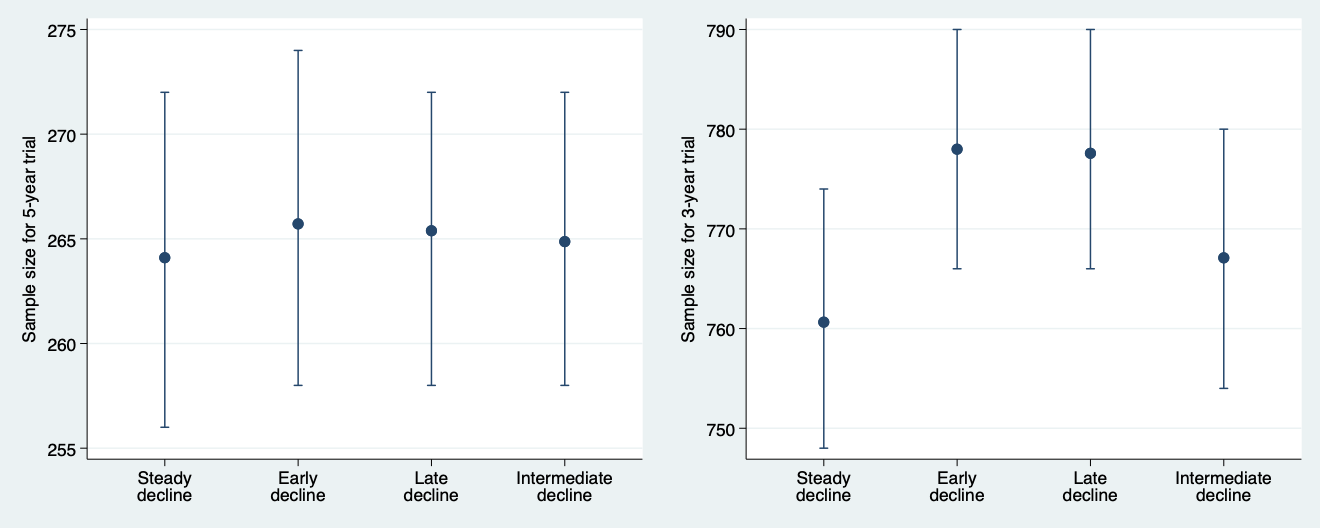


Figure A8.2.1: Mean sample sizes and IQR predicted by observational studies for 5-year trial (left) and 3-year trial (right)


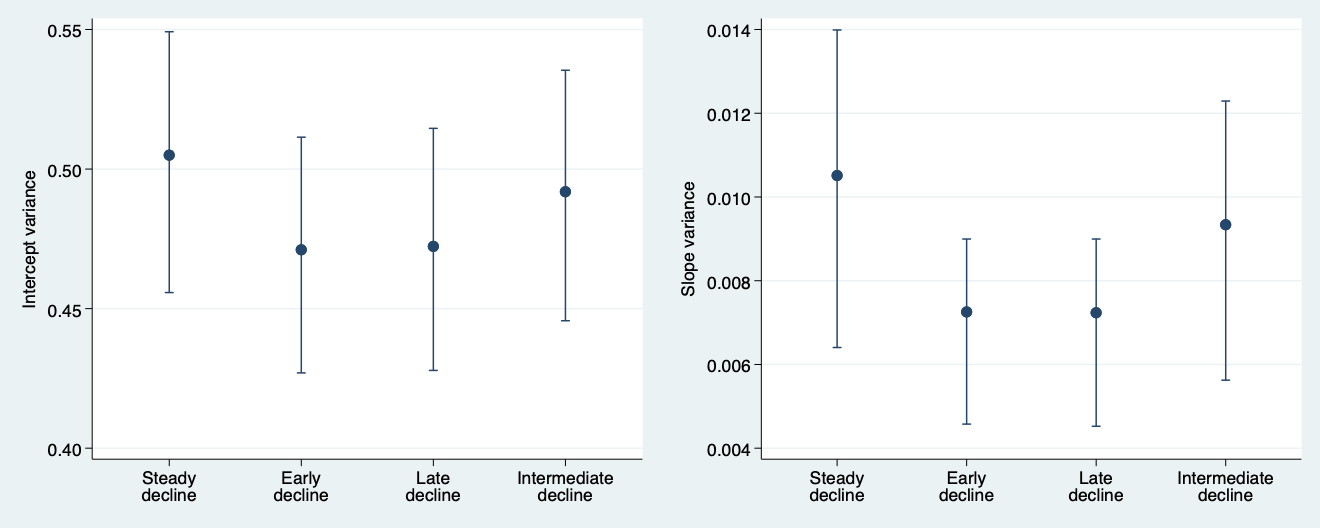


Figure A8.2.2: Mean intercept (left) and slope (right-hand panel) variance and IQR predicted by observational studies


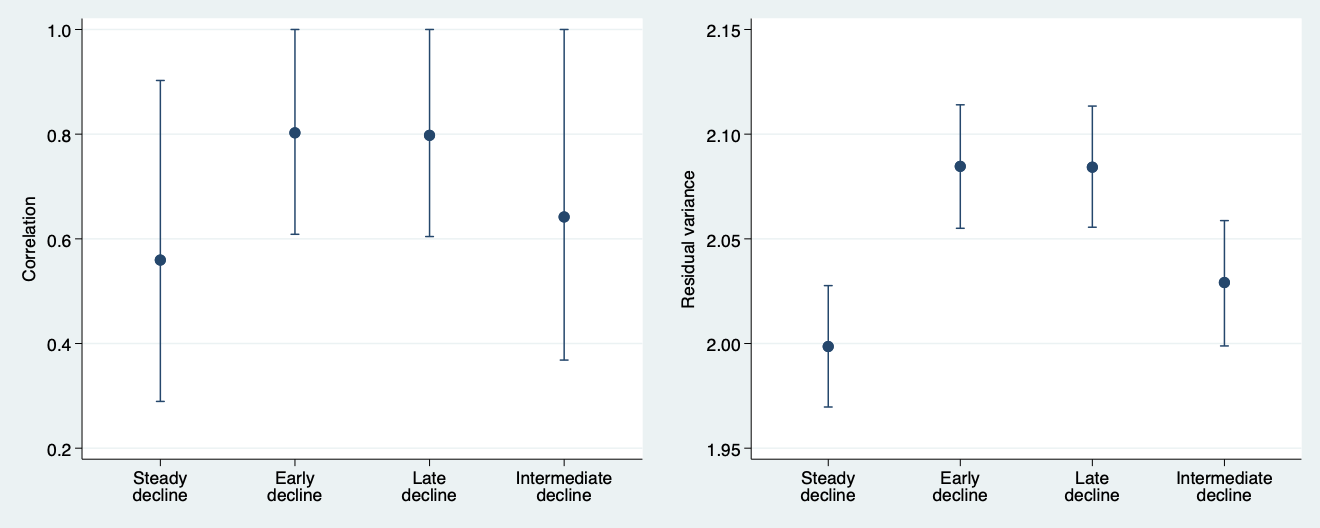


Figure A8.2.3: Mean correlation between random intercepts and slopes (left-hand panel) and mean residual variance (right-hand panel) and IQR predicted by observational studies; larger residual variance

### A9. Stata code

In this section we give minimal example Stata code for generating and analysing simulated data. Examples are given for the smaller residual error variance, steady decline trajectory, a 5-year trial and a proportional treatment effect.

#### A9.1. Generating and analysing observational study data

* size of observational study

scalar N_obs_st = 1000

* power

scalar sc_pow = 0.8

* 1- type 1 error/2

scalar sc_T1 = 0.975

* target treatment difference – can change this to -0.1 for larger residual error variance scenarios

scalar sc_tte = -0.05

* variance components for random effects

scalar sc_var_u0 = 0.5

scalar sc_var_u1 = 0.01

scalar sc_corr_u01 = 0.5

* residual error variance – can change this to 2 for larger residual error variance scenarios

scalar sc_var_e = 0.15

* open file to save sample sizes in

cap file close sampsi_file

file open sampsi_file using obs_study_steady_decline_sampsi.txt, write replace

file write sampsi_file “loop” _tab “n_5yr” _tab “n_3yr” _n

* program for drawing variables for observational study, using scalars above

cap prog drop obs_data

program obs_data

qui: drawnorm u0 u1, n(`=N_obs_st') sds(`=`=sc_var_u0'^0.5', `=`=sc_var_u1'^0.5') ///

corr(1, `=sc_corr_u01' \ `=sc_corr_u01', 1)

* person ID

gen id = _n

* expand to baseline + five annual follow-up visits

qui: expand 6

bys id: gen t = _n-1

* residual error

gen e = rnormal(0, `=sc_var_e'^.5)

end

forval i = 1/5000 {

* Generate data from observational study

* create variables

clear

obs_data

* steady decline trajectory – can change this for other trajectories

gen y = 6 + 0.2 * t + u0 + u1 * t + e

* Analyse data from observational study using random slopes model

cap: mixed y c.t || id: t, cov(uns) reml iter(100)

* save model parameters and variances

if _rc == 0 & e(converged) == 1 {

matrix Mb = e(b)

}

else {

* if doesn't converge using default settings, try using DFP algorithm

cap: mixed y c.t || id: t, cov(uns) reml iter(100) technique(dfp)

if _rc == 0 & e(converged) == 1 {

matrix Mb = e(b)

}

else {

* if still doesn't converge, try BFGS algorithm

cap: mixed y c.t || id: t, cov(uns) reml iter(100) technique(bfgs)

if _rc == 0 & e(converged) == 1 {

matrix Mb = e(b)

}

else {

* if still doesn't converge, try using matlog option

cap: mixed y c.t || id: t, cov(uns) reml iter(100) matlog

if _rc == 0 & e(converged) == 1 {

matrix Mb = e(b)

}

}

}

}

*

* Use parameters from mixed models to predict sample size for a 5-year trial

*

if Mb[1,1] != . {

* 2 person trial with first off treatment and second on treatment

matrix MX = (1, 0, 0 \ 1, 1, 0 \ 1, 2, 0 \ 1, 3, 0 \ 1, 4, 0 \ 1, 5, 0 \ ///

1, 0, 0 \ 1, 1, 1 \ 1, 2, 2 \ 1, 3, 3 \ 1, 4, 4 \ 1, 5, 5)

matrix MZ_p = (1, 0 \ 1, 1 \ 1, 2 \ 1, 3 \ 1, 4 \ 1, 5)

matrix MG_p = (exp(2*Mb[1,4]), ///

(exp(2*Mb[1,5])-1)/(exp(2*Mb[1,5])+1)*exp(Mb[1,3])*exp(Mb[1,4]) \ ///

(exp(2*Mb[1,5])-1)/(exp(2*Mb[1,5])+1)*exp(Mb[1,3])*exp(Mb[1,4]), ///

exp(2*Mb[1,3]))

matrix MSig_p = MZ_p * MG_p * MZ_p' + exp(2*Mb[1,6]) * I(6)

matrix MSigma = I(2) # MSig_p

matrix MSig_inv = inv(MSigma)

matrix MV = inv(MX' * MSig_inv * MX)

scalar sc_var_beta = MV[3,3]

scalar sc_N_5yr = ///

2*ceil(((invnormal(sc_T1)+invnormal(sc_pow))*sc_var_beta^.5/sc_tte)^2)

file write sampsi_file "`i'" _tab (sc_N_5yr) _tab

*

* Use parameters from mixed models to predict sample size for a 3-year trial

*

* 2 person trial with first off treatment and second on treatment

matrix MX = (1, 0, 0 \ 1, 1, 0 \ 1, 2, 0 \ 1, 3, 0 \ ///

1, 0, 0 \ 1, 1, 1 \ 1, 2, 2 \ 1, 3, 3)

matrix MZ_p = (1, 0 \ 1, 1 \ 1, 2 \ 1, 3)

matrix MG_p = (exp(2*Mb[1,4]), ///

(exp(2*Mb[1,5])-1)/(exp(2*Mb[1,5])+1)*exp(Mb[1,3])*exp(Mb[1,4]) \ ///

(exp(2*Mb[1,5])-1)/(exp(2*Mb[1,5])+1)*exp(Mb[1,3])*exp(Mb[1,4]), ///

exp(2*Mb[1,3]))

matrix MSig_p = MZ_p * MG_p * MZ_p' + exp(2*Mb[1,6]) * I(4)

matrix MSigma = I(2) # MSig_p

matrix MSig_inv = inv(MSigma)

matrix MV = inv(MX' * MSig_inv * MX)

scalar sc_var_beta = MV[3,3]

scalar sc_N_3yr = ///

2*ceil(((invnormal(sc_T1)+invnormal(sc_pow))*sc_var_beta^.5/sc_tte)^2)

file write sampsi_file (sc_N_3yr) _n

}

else {

file write sampsi_file "`i'" _tab "." _tab "." _n

}

}

file close sampsi_file

#### A9.2 Generating and analysing trial data

* actual treatment effect in trial – can change this for larger residual error variance scenario

scalar sc_trt = -0.05

* variance components for random effects

scalar sc_var_u0 = 0.5

scalar sc_var_u1 = 0.01

scalar sc_corr_u01 = 0.5

* residual error variance – can change this for larger residual error variance scenario

scalar sc_var_e = 0.15

* program to read in sample size for each loop

cap prog drop read_sampsi

prog read_sampsi

preserve

import delimited using obs_study_steady_decline_sampsi.txt, clear

scalar sc_N = n_5yr[`1']

* `1' = `i', i.e. loop number

restore

end

* program for generating variables to create outcome for a 5 year RCT

cap: prog drop rct_data

prog rct_data

* draw person-level random effects from multivariate normal

clear

qui: drawnorm u0 u1, n(`=`1'') sds(`=sc_var_u0^0.5', `=sc_var_u1^0.5') ///

corr(1, `=sc_corr_u01' \ `=sc_corr_u01', 1)

* `1' = number of people in trial (e.g. sc_N)

* person ID

gen id = _n

* randomise to receive treatment or not

gen rand = rnormal()

sort rand

gen trt = (_n > `=`1'/2')

drop rand

sort id

* expand to baseline + annual follow-up visits – can change this for a 3-year trial

qui: expand 6

bys id: gen t = _n-1

* residual error

gen e = rnormal(0, `=sc_var_e^.5')

end

* program for analysis of RCT data

cap prog drop rct_analysis

prog rct_analysis

* analyse trial using random slopes model

cap: mixed y c.t c.t#i.trt || id: t, cov(uns) reml iter(100) dfmethod(kroger)

cap: test c.t#1.trt, small

* save model parameters and variances

if _rc == 0 & e(converged) == 1 {

mat Mb = e(b)

}

else {

* try fitting with DFP algorithm instead

cap: mixed y c.t c.t#i.trt || id: t, ///

cov(uns) reml iter(100) technique(dfp) dfmethod(kroger)

cap: test c.t#1.trt, small

if _rc == 0 & e(converged) == 1 {

mat Mb = e(b)

}

else {

* try BFGS

cap: mixed y c.t c.t#i.trt || id: t, ///

cov(uns) reml iter(100) technique(bfgs) dfmethod(kroger)

cap: test c.t#1.trt, small

if _rc == 0 & e(converged) == 1 {

mat Mb = e(b)

}

else {

* try matlog

cap: mixed y c.t c.t#i.trt || id: t, ///

cov(uns) reml iter(100) matlog dfmethod(kroger)

cap: test c.t#1.trt, small

if _rc == 0 & e(converged) == 1 {

mat Mb = e(b)

}

else {

* set to missing if still not converged

matrix Mb = J(1, 8, .)

}

}

}

}

* analyse trial using linear trajectories, free covariance model

cap: mixed y c.t c.t#i.trt || id: , res(uns, t(t)) nocons reml iter(100) dfmethod(kroger)

cap: test c.t#1.trt, small

* save model parameters and variances

if _rc == 0 & e(converged) == 1 {

mat Mb = e(b)

}

else {

* try DFP

cap: mixed y c.t c.t#i.trt || id: , ///

res(uns, t(t)) nocons reml iter(100) technique(dfp) dfmethod(kroger)

cap: test c.t#1.trt, small

if _rc == 0 & e(converged) == 1{

mat Mb = e(b)

}

else {

* try BFGS

cap: mixed y c.t c.t#i.trt || id: , ///

res(uns, t(t)) nocons reml iter(100) technique(bfgs) dfmethod(kroger)

cap: test c.t#1.trt, small

if _rc == 0 & e(converged) == 1 {

mat Mb = e(b)

}

else {

* try matlog

cap: mixed y c.t c.t#i.trt || id: , ///

res(uns, t(t)) nocons reml iter(100) matlog dfmethod(kroger)

cap: test c.t#1.trt, small

if _rc == 0 & e(converged) == 1 {

mat Mb = e(b)

}

else {

* set to missing if still not converged

matrix Mb = J(1, 25, .)

}

}

}

}

* analyse trial using free control-group trajectory, free covariance model

cap: mixed y i.t c.t#i.trt || id: , res(uns, t(t)) nocons reml iter(100) dfmethod(kroger)

cap: test c.t#0.trt, small

* save model parameters and variances

if _rc == 0 & e(converged) == 1 {

mat Mb = e(b)

}

else {

* try DFP

cap: mixed y i.t c.t#i.trt || id: , ///

res(uns, t(t)) nocons reml iter(100) technique(dfp) dfmethod(kroger)

cap: test c.t#0.trt, small

if _rc == 0 & e(converged) == 1{

mat Mb = e(b)

}

else {

* try BFGS

cap: mixed y i.t c.t#i.trt || id: , ///

res(uns, t(t)) nocons reml iter(100) technique(bfgs) dfmethod(kroger)

cap: test c.t#0.trt, small

if _rc == 0 & e(converged) == 1 {

mat Mb = e(b)

}

else {

* try matlog

cap: mixed y i.t c.t#i.trt || id: , ///

res(uns, t(t)) nocons reml iter(100) matlog dfmethod(kroger)

cap: test c.t#0.trt, small

if _rc == 0 & e(converged) == 1 {

mat Mb = e(b)

}

else {

* set to missing if still not converged

matrix Mb = J(1, 25, .)

}

}

}

}

* analyse using mixed model with free trajectories, free covariance model

qui: gen t_trt = 0

qui: replace t_trt = t if trt == 1 & t != 0

cap: mixed y i.t i.t_trt || id: , ///

residuals(uns, t(t)) nocons reml iter(100) dfmethod(kroger)

* save model parameters and variances

if _rc == 0 & e(converged) == 1 {

mat Mb = e(b)

}

else {

* try DFP

cap: mixed y i.t i.t_trt || id: , ///

residuals(uns, t(t)) nocons reml iter(100) technique(dfp) dfmethod(kroger)

if _rc == 0 & e(converged) == 1 {

mat Mb = e(b)

}

else {

* try BFGS

cap: mixed y i.t i.t_trt || id: , ///

residuals(uns, t(t)) nocons reml iter(100) technique(bfgs) ///

dfmethod(kroger)

if _rc == 0 & e(converged) == 1 {

mat Mb = e(b)

}

else {

* try matlog

cap: mixed y i.t i.t_trt || id: , ///

residuals(uns, t(t)) nocons reml iter(100) matlog dfmethod(kroger)

if _rc == 0 & e(converged) == 1 {

mat Mb = e(b)

}

else {

* set to missing if still not converged

matrix Mb = J(1, 34, .)

}

}

}

}

if Mb[1,1] != . {

cap: test 5.t_trt, small

}

end

forval i = 1/5000 {

* Generate data from a clinical trial with the sample size predicted by observational study

read_sampsi `i'

if sc_N != . {

rct_data sc_N

* steady decline trajectory – can change this for other trajectories

gen y = 6 + 0.2 * t + sc_trt * t * trt + u0 + u1 * t + e

drop u0 u1 e

* Analyse clinical trial

rct_analysis

}

}
